# A Novel Role for FERM Domain-Containing Protein 3 in CKD

**DOI:** 10.34067/KID.0000000602

**Published:** 2024-10-16

**Authors:** Ciarán Kennedy, Ross Doyle, Oisin Gough, Caitriona Mcevoy, Susan McAnallen, Maria Hughes, Xin Sheng, Bianca Crifo, Darrell Andrews, Andrew Gaffney, Javier Rodriguez, Susan Kennedy, Eugene Dillon, Daniel Crean, Weijia Zhang, Zhengzi Yi, Viji Nair, Katalin Susztak, Joel Hirschhorn, Jose Florez, Per-Henrik Groop, Niina Sandholm, Matthias Kretzler, Gareth J. McKay, Amy Jayne McKnight, Alexander P. Maxwell, David Matallanas, Anthony Dorman, Finian Martin, Peter J. Conlon, Denise M. Sadlier, Eoin Brennan, Catherine Godson

**Affiliations:** 1 UCD Diabetes Complications Research Centre, UCD School of Medicine, Conway Institute, University College Dublin, Dublin, Ireland; 2 School of Medicine, Mater Misericordiae University Hospital, University College Dublin, Dublin, Ireland; 3 Tallaght University Hospital, Dublin; and Trinity Kidney Centre, School of Medicine, Trinity College Dublin, Dublin, Ireland; 4 Department of Medicine, University of Pennsylvania, Philadelphia, Pennsylvania; 5 Liangzhu Laboratory, Zhejiang University, Hangzhou, China; 6 Department of Nephrology, The Children's Hospital, Zhejiang University School of Medicine and National Clinical Research Center for Child Health, Hangzhou, China; 7 School of Biomolecular and Biomedical Science, Conway Institute of Biomolecular and Biomedical Research, University College Dublin, Dublin, Ireland; 8 Systems Biology Ireland, University College Dublin, Dublin, Ireland; 9 TriviumVet, Waterford, Ireland; 10 UCD Conway Institute Core Technologies, University College Dublin, Dublin, Ireland; 11 School of Veterinary Medicine, University College Dublin, Dublin, Ireland; 12 Icahn School of Medicine at Mount Sinai, New York, New York; 13 Division of Nephrology, Department of Internal Medicine, University of Michigan, Ann Arbor, Michigan; 14 Endocrine Division and Diabetes Unit, Center for Genomic Medicine, Massachusetts General Hospital, Boston, Massachusetts; 15 Broad Institute of MIT and Harvard, Cambridge, Massachusetts; 16 Folkhälsan Institute of Genetics, Folkhälsan Research Center, Helsinki, Finland; 17 Research Program for Clinical and Molecular Metabolism, Faculty of Medicine, University of Helsinki, Helsinki, Finland; 18 Department of Nephrology, University of Helsinki and Helsinki University Hospital, Helsinki, Finland; 19 Department of Diabetes, Central Clinical School, Monash University, Melbourne, Victoria, Australia; 20 Centre for Public Health, Queens University of Belfast, Northern Ireland, United Kingdom; 21 Department of Pathology, Beaumont Hospital, Dublin, Ireland; 22 National Kidney Transplant Service, Department of Nephrology and Kidney Transplantation, Beaumont Hospital, Royal College of Surgeons in Ireland, Dublin, Ireland

**Keywords:** CKD, gene expression, genetics and development, progression, proximal tubule, transcriptional profiling, tubular epithelium, tubulointerstitial disease

## Abstract

**Key Points:**

- We have identified a transcriptional signature of 93 genes associated with CKD severity and progression.
- Protein 4.1, ezrin, radixin, moesin domain-containing protein 3 gene expression is reduced in the context of more severe kidney disease and in individuals who go on to develop progressive disease.
- Protein 4.1, ezrin, radixin, moesin domain-containing protein 3 interacts with proteins of the cell cytoskeleton and cell-cell junctions in proximal tubule epithelial cells.

**Background:**

Currently, there are limited methods to link disease severity and risk of disease progression in CKD. To better understand this potential relationship, we interrogated the renal transcriptomic profile of individuals with CKD with measures of CKD severity and identified protein 4.1, ezrin, radixin, moesin-domain containing protein 3 (*FRMD3*) as a candidate gene for follow-up study.

**Methods:**

RNA-sequencing was used to profile the transcriptome of CKD biopsies from the North Dublin Renal BioBank, the results of which were correlated with clinical parameters. The potential function of FRMD3 was explored by interrogating the FRMD3 interactome and assessing the effect of lentiviral mediated FRMD3 knock down on human renal proximal tubule epithelial cells by assessing cell viability, metabolic activity, and structural markers.

**Results:**

We identified a subset of 93 genes which are significantly correlated with eGFR and percentage tubulointerstitial fibrosis at time of biopsy and with CKD progression 5 years postbiopsy. These results were validated against transcriptomic data from an external cohort of 432 nephrectomy samples. One of the top-ranking genes from this subset, FRMD3, has previously been associated with the risk of developing diabetic kidney disease. Interrogating the interactome of FRMD3 in tubule epithelial cells revealed interactions with cytoskeletal components of cell-cell junctions. Knockdown of FRMD3 expression in tubule epithelial cells resulted in increased proapoptotic activity within the cells, as well as dysregulation of E-Cadherin.

**Conclusions:**

We have identified a panel of kidney-specific transcripts correlated with severity and progression of kidney disease, and from this, we have identified a possible role for FRMD3 in tubule cell structure and health.

## Visual Abstract

**Figure:**
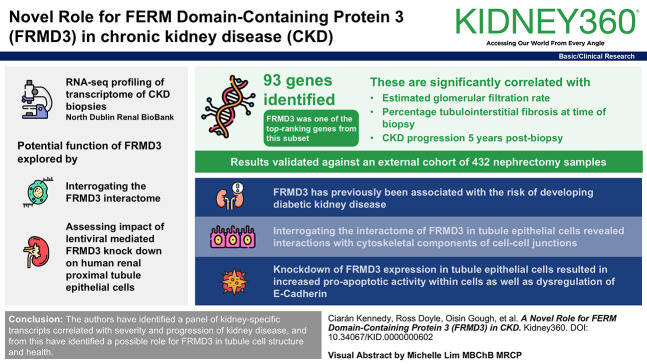

## Introduction

CKD is an insidious challenge, with risk factors for development and progression including age, obesity, cardiovascular disease, diabetes, and hypertension.^1,2^ While CKD is readily identified using simple, albeit insensitive, clinical tests, it is difficult to predict individuals at high risk of progression.^3^ Numerous genetic loci have been identified contributing to the risk of developing CKD through genome-wide association studies (GWAS), but understanding of the genetic, transcriptomic, and regulatory interactions associated with these variants and their link with CKD remains incomplete.^4,5^ To this end, transcriptomic analysis of biopsies from individuals with CKD represents a useful tool for the identification of novel drivers and potential therapeutic interventions for CKD.^6,7^

The aim of this study was to interrogate the transcriptome of kidney biopsies from a cohort of individuals with CKD, correlate them with routine clinical variables and disease progression 5 years postbiopsy, and identify key pathways implicated in disease severity and progression. We identified 93 genes commonly associated with eGFR, percentage tubulointerstitial fibrosis (%TIF), and disease progression. Among the lead candidates, we show reduced expression of protein 4.1, ezrin, radixin, moesin (FERM) domain-containing protein 3 (FRMD3) associated with progressive CKD. FRMD3 encodes FERM domain-containing protein 3, a single-pass transmembrane protein and part of the FERM domain family (4.1, ezrin, radixin, moesin).^8^ FERM domain proteins contribute to the stability of cytoskeleton-plasma membrane interactions and play a role in maintaining cell shape and integrity.^9,10^ Single nucleotide polymorphisms associated with this gene have been linked to the development of diabetic kidney disease (DKD) in GWAS from multiple cohorts.^11–13^ Our data provide a putative functional link between FRMD3 and maintenance of homeostasis and junctional integrity in the kidney tubule.

## Methods

Detailed methods are available in Supplemental Material. This study was performed in adherence to the Declaration of Helsinki, and ethical approval for this work was granted by the Beaumont Hospital Ethics (Medical Research) Committee. All participants in this study provided informed written consent before enrollment, and all data were processed in line with the General Data Protection Regulation of the European Union.

### Human Kidney Biopsies

Human kidney biopsies and baseline clinical data were provided by the North Dublin Renal Biobank (NDRBB). eGFR was calculated using the CKD Epidemiology Collaboration equation.^14^ %TIF was determined by a kidney pathologist during routine processing after biopsy and after trichrome staining and verified by a second pathologist blinded to the results of the first analysis using ImageJ for quantification. Percent glomerulosclerosis was defined as an aggregate of the percentage of sclerosed glomeruli per field. Progressive disease was defined as a doubling of serum creatinine or reaching ESKD; stable disease was defined as having a follow-up serum creatinine within 10% of enrollment value.

### Bulk RNA-Sequencing Transcriptome Analysis

mRNA from kidney biopsy samples was sequenced and aligned to the human genome assembly hg19. A linear regression model was used to examine the correlation between normalized counts, eGFR and %TIF, with age and sex used as covariables. Differentially expressed transcripts in progressive versus stable CKD were identified using the limma package^15^ in R. RNA-Sequencing (RNA-seq) data are deposited at the Gene Expression Omnibus (GSE137570).^16^

### Cell Culture

Human proximal tubule epithelial (HK-2) cells (American type culture collection) were used for *in vitro* testing in this study because this proximal tubule cell line expresses similar levels of FRMD3 at the transcript level as primary human cells in multiple publicly available datasets, while other commonly used human tubule lines express much lower levels.^17^ Lentiviral transduction was used to generate stable HK-2 lines expressing V5-tagged FRMD3, empty vector control, short hairpin RNA (shRNA) targeting FRMD3, or nontargeting control shRNA.

### Mass Spectrometry Proteomics Analysis

Affinity Purification Mass Spectrometry was used to interrogate the FRMD3-V5 interactome. Proteomics data were deposited using the PRoteomics IDEntifications database partner repository (PXD015297).

### *In Vitro* Functional Assays

Cell viability, oxygen consumption, and caspase 3/7 activity were assessed. Protein levels were detected by immunoblotting and immunocytochemistry as appropriate. mRNA levels were assayed by quantitative PCR.

## Results

### Identification of Kidney Transcripts Associated with CKD Severity at Biopsy

The transcriptomes of individuals from the NDRBB (cohort 1, *n*=24) were used to investigate the link between gene expression and CKD severity. As anticipated, strong correlation was observed between %TIF and glomerulosclerosis on biopsy (Pearson *r*=0.75, *P* = 1.001×10^−9^; Supplemental Figure 1A) and strong negative correlation between %TIF and eGFR at time of biopsy (Pearson *r*=−0.61, *P* = 5.274×10^−6^; Supplemental Figure 1B), as well as between eGFR and glomerulosclerosis (Pearson *r*=−0.45, *P* = 0.0016; Supplemental Figure 1C).

**Figure 1:**
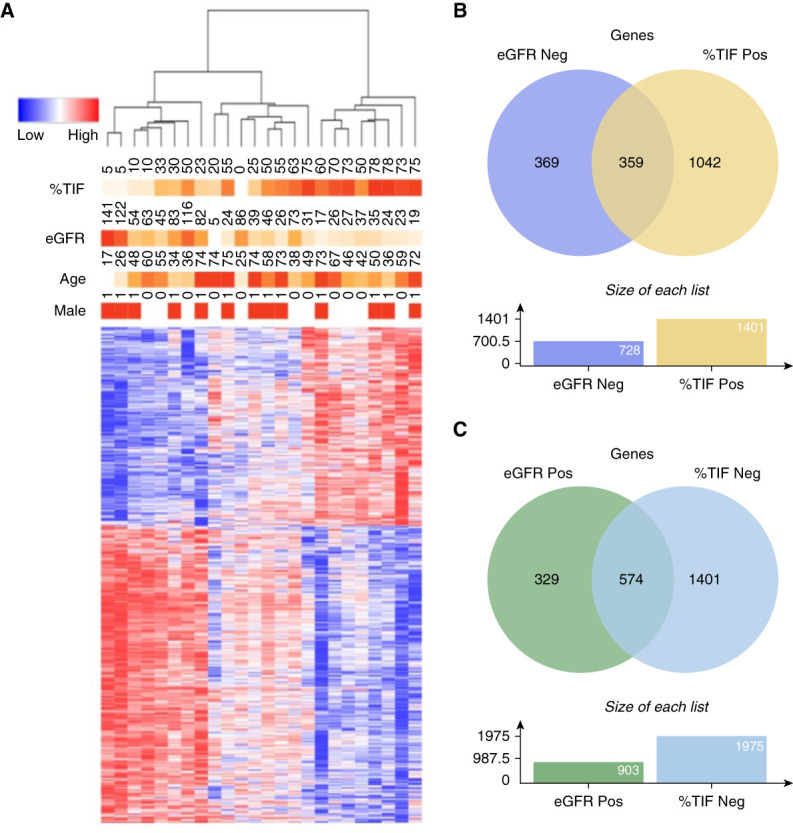
**Transcriptomic analysis of human kidney biopsy samples links gene expression and disease severity.** (A) Heatmap of gene expression in human kidney biopsy samples from the NDRBB and eGFR, as well as %TIF, with 935 genes correlating with both in our cohort (*n*=24). Of these, 359 genes showed higher expression in the setting of more severe disease (B), being eGFR Neg and %TIF Pos, and 574 genes showed higher expression in the setting of less severe disease (C), being eGFR Pos and %TIF Neg. eGFR Neg, negatively correlated with eGFR; eGFR Pos, positively correlated with eGFR; NDRBB, North Dublin Renal Biobank; %TIF Neg, negatively correlated with %TIF; %TIF, percentage tubulointerstitial fibrosis; %TIF Pos, positively correlated with %TIF.

Correlation analysis was then used to link transcriptomic profiles of the biopsied individuals to both eGFR and %TIF (Figure 1). A total of 1631 genes showed statistically significant correlation with eGFR, 903 genes positively correlated, and 728 genes negatively correlated (*P*_adj_ < 0.05, Supplemental Table 2). A larger number of genes, 3376 in total, were significantly correlated with %TIF, with 1401 positively correlated and 1975 negatively correlated (*P*_adj_ < 0.05, Supplemental Table 3). Interrogating these gene sets against the Go Biological Processes and Reactome databases revealed that genes positively correlated with eGFR or negatively with %TIF showed significant enrichment for mitochondrial processes and oxidative phosphorylation (Supplemental Figures 2 and 3 and Supplemental Tables 4–7). Notably one of the most highly enriched of these processes is that of fatty acid beta oxidation in genes negatively correlated with %TIF and positively correlated with eGFR (fatty acid beta oxidation [GO:0006635], *q* value = 0.01533, combined score=47.1). These findings are in keeping with the critical role mitochondrial health plays in kidney function and kidney disease^18,19^ and supports previously published findings that defective fatty acid oxidation is a key driver of fibrosis in the kidney epithelium.^20^ Genes negatively correlated with eGFR or positively with %TIF showed overlapping enrichment in immune system and cytokine signaling (Figure 1B, Supplemental Figures 2 and 3, and Supplemental Tables 4–7).

### Association of Kidney Transcripts with CKD Progression

The renal transcriptomic profile of individuals with progressive CKD was compared with those whose CKD remained stable at a 5-year follow-up. Transcriptomic profiles were generated from a second cohort of individuals (Cohort 2, *n*=17) with a median follow-up of 60 months (Table 1 and Supplemental Table 1). Biopsied individuals were classified as either having progressive CKD (*n*=8) or as having stable CKD (*n*=9). Nine hundred ninety-eight transcripts were identified as being differentially expressed between both groups, with 660 transcripts upregulated and 338 transcripts downregulated in those experiencing progressive CKD, after adjustment for age, sex, and setting an false discovery rate of <0.05 (Supplemental Table 8).

**Table 1:** Demographics of study participants

Demographic Categories	Cohort 1 (*n*=24)	Cohort 2 (*n*=17)	Cohort 2 - Progressive CKD (*n*=8)	Cohort 2 - Stable CKD (*n*=9)
Male/female, %	54/46	47/53	63/37	33/67
**Comorbidities at time of biopsy, %**				
Hypertension	67	76	100	56
Diabetes	13	0	0	0
Dyslipidemia	33	35	38	33
**Prescribed medications at time of biopsy, %**				
RAASi (ACEi or ARB)	67	47	50	44
CCB	21	65	88	44
Thiazide or loop diuretic	29	47	63	33
**Clinical variables at time of biopsy, median (IQR)**				
Age (yr)	50 (37–69)	48 (45–67)	58 (41–72)	48 (45–59)
BMI (kg/m^2^)	27 (25–32)	27 (24–30)	29 (26–34)	24 (23–28)
Systolic BP (mm Hg)	132 (121–141)	137 (122–151)	137 (130–142)	137 (117–155)
Diastolic BP (mm Hg)	75 (68–78)	85 (69–89)	80 (65–86)	85 (70–89)
Hemoglobin (g/dl)	12 (11–14)	12 (11–13)	11 (11–12)	13 (11–14)
White blood cell count (×10^9^)	7 (6–8)	7 (5–8)	7 (5–8)	7 (5–9)
Platelets (×10^9^)	250 (209–238)	238 (232–259)	257 (211–274)	236 (235–258)
Albumin (g/dl)	39 (32–41)	38 (33–40)	35 (33–39)	39 (34–43)
Tubulointerstitial fibrosis (%) on biopsy	50 (18–71)	35 (20–70)	70 (56–74)	20 (13–22)
eGFR at time of biopsy (ml/min per 1.73 m^2^)	32 (19–65)	29 (16–60)	16 (9–19)	60 (46–93)
eGFR at 24 mo follow-up (ml/min per 1.73 m^9^)	52 (37–80)	42 (13–57)	9 (5–14)	55 (42–84)

ACEi, angiotensin-converting enzyme inhibitor; ARB, angiotensin receptor blocker; BMI, body mass index; CCB, calcium channel blocker; IQR, interquartile range; RAASi, renin-angiotensin-aldosterone system inhibitor.

**Figure 2:**
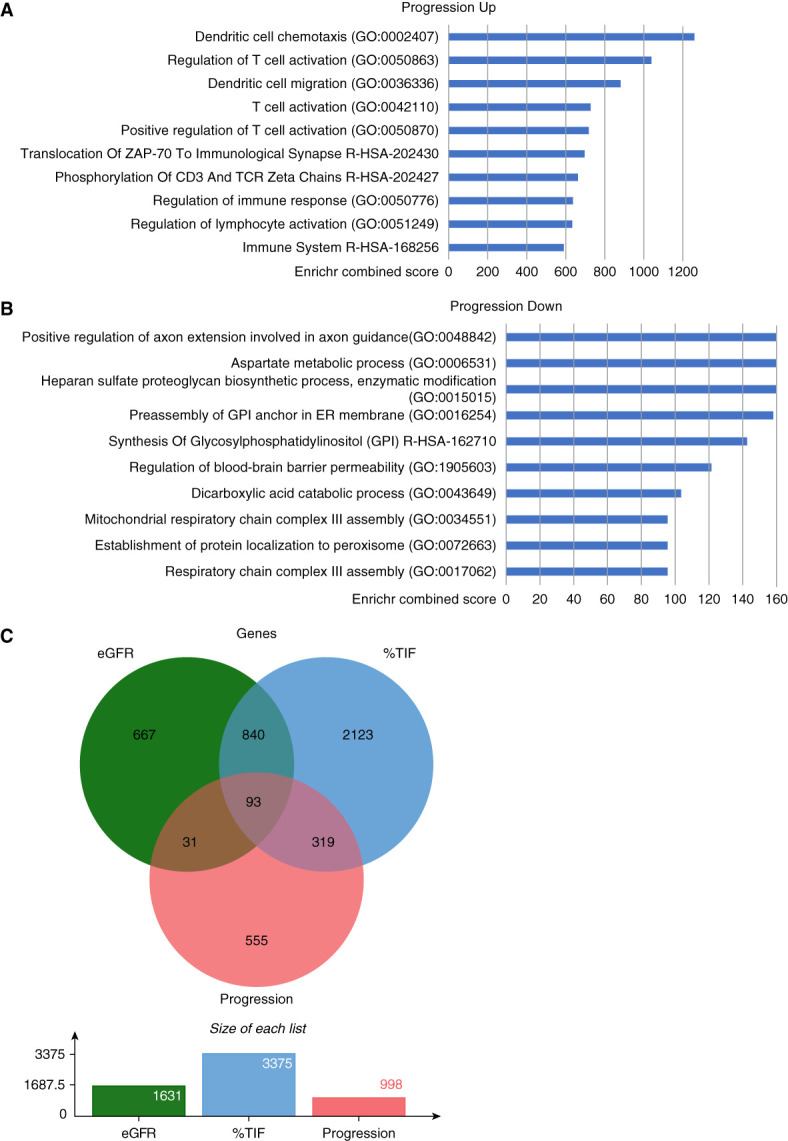
**Differential expression analysis of progressive versus stable CKD highlights key dysregulated processes and displays overlap with markers of disease severity.** Gene set enrichment analysis of genes differentially expressed within patients with progressive CKD versus Stable CKD was performed using the Enrichr platform and the Reactome and Go Biological Processes databases with the top 50 processes from each dataset by combined score used for visualization and analysis. Genes upregulated in the progressive CKD group (A) were enriched for immune system-related terms, while those downregulated in the progressive CKD group (B) were enriched for cell and mitochondrial metabolic processes. (C) Comparing genes which correlate with eGFR and %TIF in either direction with genes which are differentially expressed between the progressive CKD group and the stable CKD group reveals 93 genes which were common across all three analyses. ER, endoplasmic reticulum; GPI, glycosylphosphatidylinositol.

These gene lists were interrogated using Enrichr-KG platform (Go Biological Processes and Reactome databases) (Figure 2, A and B). Genes upregulated in the progressive CKD group were highly enriched in immune system and inflammation related terms (Supplemental Table 9). Many of these enriched terms overlap with those from genes positively correlated with %TIF, with 55 of the top 100 enriched terms being identical (Supplemental Figure 3C), compared with just eight of the top 100 enriched terms for genes negatively correlated with eGFR.

We investigated the overlap between genes correlating with eGFR and %TIF at time of biopsy in Cohort 1 with genes differentially expressed in progressive CKD versus stable CKD in Cohort 2. A total of 93 genes met these criteria (Figure 2C and Supplemental Table 10), with 68 genes upregulated in the progressive CKD group and associated with more severe disease (correlating negatively with eGFR and positively with %TIF), while 27 genes were downregulated in the progressive CKD group and associated with less severe disease (positive correlation with eGFR and negative with %TIF). These 93 genes were further validated using transcriptomic data from a cohort of 432 microdissected surgical nephrectomies.^21,22^ Within this dataset, data were available for 82 of these 93 genes, of which 76 were significantly correlated with eGFR (*P* < 0.05) and all 82 were correlated with %TIF (*P* < 0.05) within the tubule compartment (Supplemental Table 11).

To prioritize candidate genes for further investigation, we ranked genes based on the degree of correlation with disease severity in this larger external cohort, in addition to differential expression between our progressive and stable CKD groups and correlation with eGFR and %TIF (Supplemental Table 12). For genes negatively associated with disease severity, and with progression, six genes ranked within the top ten genes associated with each of these categories (FRMD3, SLC2A12, TRIM50, GALM, DNMT3L, IP6K3).

FRMD3, one of the genes most negatively associated with disease severity and progression (strongest positive correlation with eGFR and strong negative correlation with %TIF in the larger external cohort, Supplemental Figure 4, Supplemental Table 11), has previously been associated with DKD risk in multiple GWAS and is speculated to contribute to FERM protein regulation of kidney function.^9–13^ Other genes of note include *SLC2A12*, which encodes the glucose transporter GLUT12, elevated in animal models of DKD and hypertension,^23,24^ and IP6K3 encoding inositol hexakisphosphate kinase 3, which regulates phosphate excretion and is downregulated in murine focal segmental glomerulosclerosis.^25,26^

### FRMD3 and Kidney Disease

**Figure 3:**
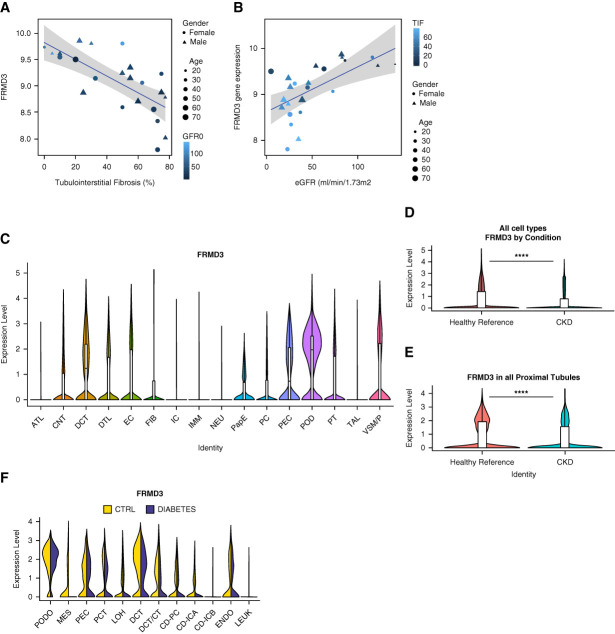
**FRMD3 expression levels are negatively associated with disease in the human kidney.** FRMD3 expression by RNA-seq inversely correlated with (A) %TIF (*r*=−0.73; *P* = 4.817×10^−5^) and positively with (B) eGFR (*r*=0.62; *P* = 0.001175) at time of biopsy in a cohort of patient kidney biopsies (*n*=24). (C) FRMD3 expression in kidney cell types by single-nuclear RNA-seq. Using a publicly available single-nuclear RNA-seq dataset of patient kidney biopsies from the Kidney Precision Medicine Project, FRMD3 expression was found to be expressed in many cell types within the kidney including tubule and glomerular cell types. (D) FRMD3 levels in the kidney precision medicine project single-nuclear RNA-sequencing dataset from healthy reference versus patients with CKD shows a reduction of FRMD3 levels averaged across all clusters in CKD versus healthy reference samples (average log_2_fold-Change −0.34, *P*_adj_ = 2.43×10^−288^) using the Wilcoxon rank-sum test with Bonferroni correction in Seurat. ****P < 0.0001. (E) FRMD3 levels in the proximal tubule cluster of the kidney precision medicine project single-nuclear RNA-sequencing dataset from healthy reference versus CKD patients shows a reduction of FRMD3 levels in CKD versus healthy reference samples (average log_2_fold-Change=−0.53, *P*_adj_ = 3.44×10^−139^) using the Wilcoxon rank-sum test with Bonferroni correction in Seurat. ****P < 0.0001. (F) Using a publicly available single-nuclear RNA-seq dataset from the Humphries laboratory, accessed at http://humphreyslab.com/SingleCell/, FRMD3 was again found to be expressed in many cell types within the kidney, with lower expression observed throughout in early diabetic nephropathy patients versus control samples. For box and whisker plots within violin plots (C–E), the box ranges from the 25th percentile to the 75th percentile, with whiskers extending 1.5 times the IQR past those points. The internal line represents the median. ATL, ascending thick limb; CD-ICA, collecting duct intercalating cell A; CD-ICB, collecting duct intercalating cell B; CD-PC, collecting duct principal cell; CNT, connecting tubule; DCT/CT, distal convoluted tubule/connecting tubule; DTL, distal thick limb; EC, endothelial cell; ENDO, endothelial cell; FIB, fibroblast; FRMD3; protein 4.1, ezrin, radixin, moesin domain-containing protein 3; IC, intercalating cell; IMM, immune cells; LEUK, leukocytes; LOH, loop of Henle; MES, mesenchymal cells; NEU, Schwann cell/neural; PapE, papillary tip epithelial cell; PC, principal cell; PCT, proximal convoluted tubule; PEC, parietal epithelial cells; PEC, parietal epithelial cell; POD, podocyte; PODO, podocyte; PT, proximal tubule; RNA-seq, RNA-sequencing; TAL, thick ascending limb; VSM/P, vascular smooth muscle/pericyte.

FRMD3 expression correlated negatively with %TIF (*r*=−0.73; *P* = 4.817×10^−5^) and positively with eGFR (*r*=0.62; *P* = 0.001) at the time of biopsy (Figure 3, A and B). FRMD3 transcript levels were downregulated in individuals with progressive versus stable CKD (log_2_fold-Change=−1.1, *P*_adj_ = 0.043) (Supplemental Table 8).

Single-nucleus RNA-seq datasets from 13 healthy reference, and ten CKD samples from the Kidney Precision Medicine Project, reveals FRMD3 expression in primarily epithelial cell types, including proximal tubules (Figure 3C), consistent with previously identified roles for FERM domain-containing proteins in the kidney.^27,28^ FRMD3 expression is significantly reduced in CKD relative to healthy controls looking at all cells (average log_2_fold-Change=−0.34, *P*_adj_ = 2.43×10^−288^) (Figure 3D and Supplemental Table 13) and specifically within the proximal tubule cluster (average log_2_fold-Change=−0.53, *P*_adj_ = 3.44×10^−139^) (Figure 3E and Supplemental Table 14).

We then interrogated a dataset of single-nuclear RNA-seq data obtained from the cortex of three nondiabetic healthy control and three diabetic participants after nephrectomy for renal mass, using the web-based Kidney Interactive Transcriptomics tool^29,30^ (http://humphreyslab.com/SingleCell/). This dataset showed a similar reduction in FRMD3 expression when comparing early stage DKD (a subtype of CKD) samples to controls (Figure 3F).

### FRMD3 Knockdown Results in Dysregulation of the Adherens Junction and Decreased Cell Viability in Proximal Tubule Epithelial Cells

**Figure 4:**
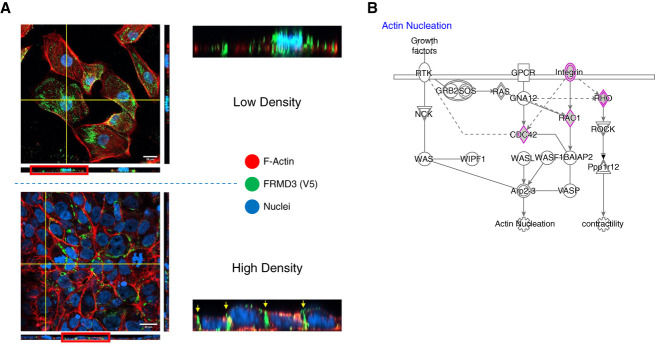
**FRMD3 is localized to cell-cell contacts in human proximal tubule cell monolayers.** V5-tagged FRMD3 overexpressing HK-2 cells were used to visualize the location of FRMD3 within the cell by laser scanning confocal microscopy z-stack (A). At low density, FRMD3 appears diffusely spread throughout the membrane of the cell, while at high density FRMD3 appears to be enriched at cell-cell contacts (yellow arrows). The main image at each density (high density: 63× magnification, low density: 40× magnification, scale bars 20 *µ*m) represents a single optical section of the sample; the yellow crosshairs represent the area taken as orthogonal slices displayed to the right (YZ) and bottom (XZ) of the main images. The red boxes around the XZ orthogonal sections mark the area enlarged to the right of both sets of images. Proteomic analysis of FRMD3-V5 interactors in these cells reveals interactions between FRMD3 and proteins linked with maintenance of the F-actin cytoskeleton (purple), including RHO-family GTPases like CDC42 and RAC1, as well as Integrin B1 (B, adapted from Ingenuity pathway analysis, Qiagen). HK-2, human proximal tubule epithelial. RHO, RAS homolog.

To investigate the function of FRMD3 in the kidney, we used immunocytochemistry to visualize FRMD3 localization in FRMD3-V5 overexpressing HK-2 cells. The observed switch in localization of FRMD3 based on degree of cell-cell contact (Figure 4 A) is consistent with other members of the FERM family where, upon activation, membrane and protein-binding sites are unmasked, allowing targeting of the protein to specific sites within the cell.^31,32^ In HK-2 cell monolayers, FRMD3 is located at the periphery of the cell, as are the tight junction marker Zo-1, the adherens junction protein p120-Catenin, and F-actin (Figure 4B and Supplemental Figure 5).

**Figure 5:**
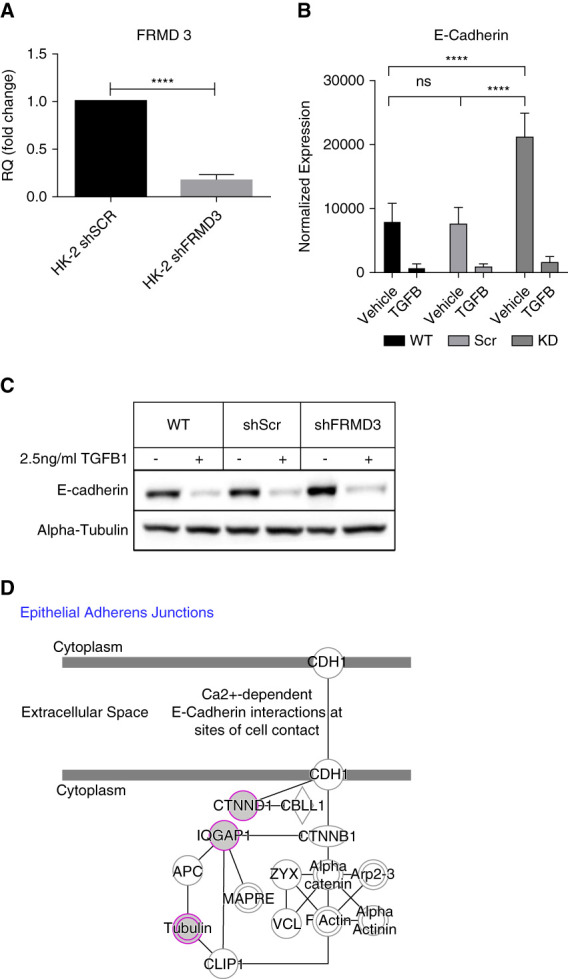
**Knockdown of FRMD3 in human proximal tubule cells disrupts cell junction protein levels.** To recapitulate the lower levels of FRMD3 seen within patients with CKD and diabetes nephritis, a HK-2 was used to generate stable shRNA knockdowns against FRMD3 or a nontargeting control shRNA. (A) The FRMD3 knockdown cells showed a 0.8228±0.030-fold reduction in FRMD3 mRNA levels compared with the scrambled non-targeting control shRNA expressing cells (****P < 0.0001, delta delta CT method, B-actin housekeeping control). (B) To investigate the role of FRMD3 within epithelial cell junctions, FRMD3 knockdown and scrambled control shRNA expressing HK-2 cells were treated with 2.5 ng/ml TGF*β*1 or vehicle for 48 hours. After this treatment, we performed Western blot against E-cadherin, a key marker of epithelial cell integrity (densitometric analysis on *n*=3 replicates normalized to *α*-tubulin expression) (C) representative western blot for (B). FRMD3 knockdown resulted in a basal 2.4-fold increase in E-cadherin levels (*P*_adj_ = 0.0001). TGF*β*1 treatment resulted in an almost 90% decrease in the amount of E-cadherin protein within the scrambled control shRNA expressing cells (88.8% reduction, *P*_adj_ = 0.0031). (D) Proteomic analysis of FRMD3 interactors in FRMD3-V5 overexpressing HK-2 cells also revealed interactions with components of the adherens cell-cell junction (purple), including the scaffold protein IQGAP1, delta1 Catenin (CTNND1), and with components of the tubulin cytoskeleton (TUBA4A, TUBA4B, TUBB, TUBB3, TUBB6, TUBB8) (adapted from ingenuity pathway analysis, Qiagen). shSCR, short hairpin scrambled control RNA; shRNA, short hairpin RNA.

We report that reduced FRMD3 expression is associated with disease severity. To model this phenotype, HK-2 cells stably expressing an shRNA targeting FRMD3 were generated by lentiviral transduction (scrambled nontargeting shRNA was used for controls). The FRMD3 knockdown cell line showed an 82% +/- 3% reduction in FRMD3 mRNA expression relative to control (Figure 5A) (*P* < 0.0001).

FRMD3 knockdown and control cells (as above) were treated with TGF*β*-1 (5 mg/ml, 48 hours). TGF*β*-1 reduced the amount of E-Cadherin protein present by almost 90% in both control and FRMD3 shRNA knockdown HK-2 cells (shScrambled: 88.8% reduction, *P*_adj_ = 0.0031, shFRMD3: 89.8% reduction, *P*_adj_ = <0.0001) when compared with vehicle-treated cells (Figure 5, B and C, full length blot images shown in Supplemental Figure 6). Intriguingly, knockdown of FRMD3 resulted in a 2.4-fold increase in E-Cadherin levels (*P*_adj_ = 0.0001) while still permitting TGF*β*-1 to reduce E-cadherin expression to near the same levels as in the control scrambled shRNA expressing cells. This suggests a disruption to the regulation of cadherins junctions, and likely cell-cell communication, exacerbated by TGF*β*-1.

**Figure 6:**
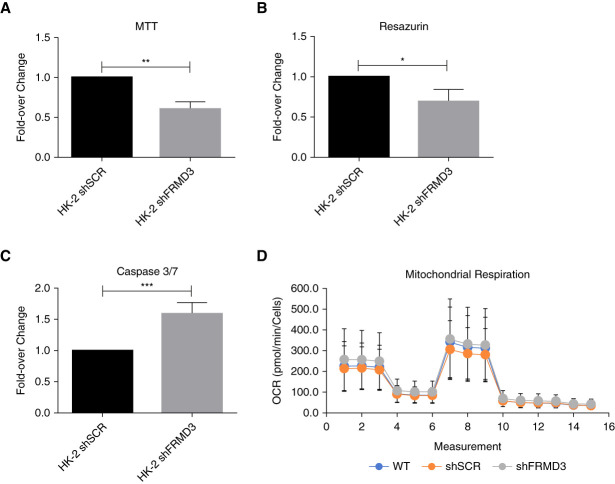
**Knockdown of FRMD3 in human proximal tubule cells results in decreased cell viability.** HK-2 expressing either a FRMD3-targeting shRNA or a control nontargeting shRNA (shSCR) were used to investigate metabolic function and viability in cells with reduced FRMD3 expression. MTT and Resazurin assays were used to assess NAD(P)H-dependent metabolic function within these cells. Knockdown of FRMD3 resulted in a 39.29%±5.244% (*P* = 0.0017) reduction in NAD(P)H metabolic activity compared with the scrambled nontargeting shRNA expressing cells (A), agreeing with the resazurin assay showing a reduction of 30.24%±8.433% (*P* = 0.0230) (B). Values were normalized to total mitochondrial content using Mitotracker Green. (C) A synthetic fluorescent substrate (synthetic fuorogenic substrate for caspase 3 (Acetylated)) was used to assess the activity of proapoptotic effector caspases (caspase 3 and caspase 7) in the FRMD3 knockdown cells, showing an increase in activity of 58.91%±8.935% (*P* = 0.0006) compared with the scrambled nontargeting control shRNA expressing cells. (D) This disruption to NAD(P)H-dependent metabolism was observed in the absence of disruption to oxygen-dependent cell metabolism as measured by the mito stress test assay using the Seahorse XF pro instrument. This implies the observed results were not because of large-scale mitochondrial dysfunction. For all, *n*=3 experiments. MTT, methylthialazole tetrazolium; NAD(P)H, dihydrogennicotinamide adenine dinucleotide phosphate; OCR, oxygen consumption rate.

Disruption of cell-cell junctions can affect homeostasis, driving changes in cellular metabolism and viability. Resazurin and methylthialazole tetrazolium assays were used to evaluate metabolic activity within these cells as an indicator of cell health. Knockdown of FRMD3 resulted in a decrease in dihydrogennicotinamide adenine dinucleotide phosphate-dependent oxidoreductase activity by methylthialazole tetrazolium assay of 39.29%±5.244% (*P* = 0.0017) and by resazurin assay, 30.24%±8.433% (*P* = 0.0230) compared with a scrambled nontargeting control shRNA (Figure 6, A and B).

Activity of proeffector caspases was measured using the fluorescent synthetic substrate synthetic fluorogenic substrate for caspase 3 (Acetylated). shRNA-mediated knockdown of FRMD3 resulted in an increased basal activity of caspase 3/7 by 58.91%±8.935% (*P* = 0.0006) relative to control cells (scrambled RNA as above) (Figure 6 C). Reduced dihydrogennicotinamide adenine dinucleotide phosphate metabolic activity and increase in proapoptotic caspase 3/7 activity were observed in the absence of any dysfunction in cell respiration by Seahorse Mito stress test assay (Figure 6D), suggesting no large-scale mitochondrial dysfunction subsequent to FRMD3 knock down.

### FRMD3 Protein-Interaction Network in Kidney Tubule Epithelial Cells

Using liquid chromatography with tandem mass spectrometry of cell lysates from HK-2 cells over expressing V-5 tagged FRMD3, we identified 244 specific interactors of FRMD3, enriched when compared with empty vector V5 expressing (FRMD3/EV ≥2, *P* < 0.05) (Supplemental Table 15). Among the top ranked interactors, we identified CKAP4 (*P* = 1.01×10^−4^), which has previously been reported to interact with FRMD3 in an affinity purification-mass spectroscopy interactome screen.^33^ Pathway analysis of these FRMD3 interactors highlighted functional enrichment for proteins related to epithelial adherens junction signaling (Supplemental Table 16). A cluster of FRMD3 protein interactants was identified as crucial to regulating E-cadherin mediated cell-cell interactions (CTNND1, IQGAP1, TUBA4A, TUBA4B, TUBB, TUBB3, TUBB6, TUBB8) (Figure 5D). Further clusters highlighted actin cytoskeleton dynamics (CDC42, RAC1, ITGB1), mitochondrial function, and proteasomal degradation.

In aggregate, these functional data complement our bioinformatic analysis linking reduced levels of FRMD3 to progressive and more severe CKD. The localization of FRMD3 to cell-cell contacts and the disruption of E-cadherin levels in the presence and absence of damaging stimuli suggests that FRMD3 contributes to the stability of the proximal tubule junction, the loss of which could potentially contribute to reduced function in CKD.

## Discussion

We have used transcriptomic analysis of kidney biopsies to build a profile of genes disrupted in CKD and investigated their correlation with both eGFR and %TIF at time of biopsy, in the setting of more severe (lower eGFR, higher %TIF) and of less severe disease (higher eGFR, lower %TIF). In a separate cohort, we built a profile of differentially expressed genes at the time of biopsy in individuals who subsequently developed progressive kidney disease versus those with stable kidney disease. These gene sets identified a signature of 93 genes significantly associated with eGFR, %TIF, and disease progression. Of these 93 genes, FRMD3 was among the genes most consistently associated with both disease severity and progression, with higher levels of FRMD3 gene expression being associated with less severe disease, as well as with being more highly expressed in stable kidney disease. Functional interrogation of FRMD3 in the HK-2 proximal tubule cell line implicated FRMD3 in the maintenance of cellular integrity.

When comparing these sets of gene expression profiles, dysregulated pathways correlating with %TIF more closely matched those differentially expressed in biopsies of individuals with progressive disease than those with stable disease (Figure 2, C and D). The results from our transcriptomic analysis in the NDRBB cohorts were also verified by comparison with transcriptomic data from the tubule compartment of a large cohort of micro dissected biopsy samples (*n*=432), which reiterated the correlation of the 93 gene signature with eGFR and %TIF (Supplemental Table 11). The stronger overlap between dysregulated processes in %TIF and disease progression may be due to greater representation of tubular cells within the biopsy samples when compared with glomerular components. The gene signatures presented here are common to a generalized CKD cohort. It is possible that with larger cohorts with distinct diagnoses disease-specific indicators of severity and or progression might be identified. It should be noted that though members in the progressive CKD group had a lower eGFR and higher %TIF at the time of biopsy than the stable CKD group (Table 1), correlation analysis of gene expression and eGFR and %TIF failed to fully capture the gene expression changes between the progressive and stable CKD groups. This suggests that the changes in gene expression observed are not wholly due to initial differences in disease severity.

Consistent with a role for inflammation in CKD,^34–36^ we observe enrichment of transcriptomic signatures associated with inflammation, immune cell function, and activation in more severe disease (Figure 2, A and B, and Supplemental Figures 1 and 2, C and D). Targeting inflammatory pathways in the context of CKD has had limited success.^37–41^ It is noteworthy that the beneficial effects of SGLT-2 inhibitors and GLP-1 agonists on renal function may reflect anti-inflammatory responses independent of regulation of blood glucose.^42–46^

Kidney function involves numerous energy-intensive processes.^42^ Mitochondrial dysfunction plays a pivotal role in the pathophysiology of both acute and CKD, and kidney phenotypes are a common manifestation of mitochondrial diseases.^18,42,43^ We report expression of gene sets related to mitochondrial function which are negatively correlated with CKD severity and downregulated in progressive versus stable CKD (Figures 2B and 4C and Supplemental Figures 1 and 2D). Among the most affected transcripts are a large cluster of dihydrogennicotinamide adenine dinucleotide:ubiquinone oxidoreductase supernumerary subunits of Mitochondrial complex I, cytochrome-c oxidase subunits of Mitochondrial complex 4, and ATP synthase subunits of Mitochondrial complex 5.

Our data suggest that downregulation of FRMD3 may play a key role in CKD severity and progression. While previous studies have strongly linked single nucleotide polymorphisms associated with FRMD3 to the risk of development of DKD, relatively little is known about its function within the cell.^11–13^ FERM domain-containing protein family members have been identified as key regulators of podocyte health.^44,45^ Published single cell transcriptomics datasets show *FRMD3* to be widely expressed in the kidney, with expression detectable in proximal and distal convoluted tubule epithelial cells, podocytes, and glomerular endothelial cells, FRMD3 expression is reduced in DKD and CKD (Figure 3, C–F).

Taking the data from our bulk RNA-seq together with these data from human single-nuclear RNA-seq datasets, it is possible that the observed inverse relationship between FRMD3 expression and CKD severity reflects both a loss of FRMD3 expression in the kidney together with a disease-driven loss of cells that that express FRMD3, *e.g*., proximal tubular epithelia and podocytes. Although many cell types within the kidney show high levels of FRMD3 expression, tubular epithelial cells were selected for further functional investigation, in part because of the higher number of these cells in the kidney compared with other cell types. It is possible that FRMD3 plays other specific roles within the kidney, for example, in podocytes which typically express high levels of FRMD3; however, this is outside the scope of this study. Further study and utilization of techniques such as single-cell RNA-seq or spatial transcriptomic analysis are required to determine cell type-specific signatured within such samples.

Proteomic analysis using V5-Tagged FRMD3 overexpressing human proximal tubule (HK-2) cells identified 244 significantly enriched FRMD3 interactors by affinity purification mass spectrometry. Pathway analysis of these enriched proteins showed an enrichment for proteins involved in cell-cell junctions and cytoskeletal arrangement, as well as for mitochondrial stability and proteasomal degradation. Although enriched interactors were identified located in the inner mitochondrial membrane, including hydroxyacyl-CoA dehydrogenase trifunctional multienzyme complex subunit alpha and hydroxyacyl-CoA dehydrogenase trifunctional multienzyme complex subunit beta components of the fatty acid beta-oxidation pathway, knockdown of FRMD3 in HK-2 cells did not show altered mitochondrial respiration or carnitine palmitoyltransferase I-dependant long-chain fatty acid oxidation by Seahorse assay (Figure 6D and Supplemental Figure 7).

FERM domain-containing proteins play an integral part in stabilizing cell structure, linking the plasma membrane with underlying cytoskeletal components. We show that FRMD3 is associated with components of the adherens junction complex at the plasma membrane, as well as with components regulating the tubulin and actin cytoskeleton at cell-cell junctions. These interactions between FRMD3 and critical components of the cytoskeleton and of adherens junctions may contribute to the links seen between FRMD3 expression and kidney disease because these junctions are crucial for maintenance of cell-cell adhesion and cell polarity.^46,47^ It is noteworthy that loss of FRMD3 expression has recently been associated with increased metastatic potential and poor prognosis in breast cancer, as well as contributing to mammary epithelial cell fate through regulation of epithelial to mesenchymal transition and cytoskeletal dynamics.^48,49^ Interestingly, similar roles and interactions have been identified within other members of the FERM domain-containing protein family.^50–54^ Many of these family members have been shown to be recruited to cell-cell junctions in different cell types, where disruption of these proteins inhibits the proper formation and regulation of these junctions. This seems to be through the association of these proteins with members of the adherens junction complex, mainly within the catenin family, and through interactions with cytoskeleton dynamics-associated small GTPases such as Rho, Rac1, and CDC42. Although these interactions have been well known for this family of proteins for some time now, the work presented here is the first time these functions have been functionally shown for FRMD3. This provides another mechanism by which loss of FRMD3 expression could contribute to the development of kidney disease, either through the interactions with junctional proteins and associated cytoskeletal proteins or through the direct interactions with Rho/Rac signaling pathway members as described in this study, which mediate the arrangement of the cytoskeleton in various kidney cell types contributing to proper barrier function of the kidney which are subsequently altered in disease.^55,56^

In summary, transcriptomic analysis of biopsies from individuals with CKD identified a signature of 93 genes associated with CKD severity and predictive of disease progression in independent cohorts. Interrogation of this signature in microarray data from a larger cohort resulted in the identification of FRMD3 as a potential target for further investigation. Our work demonstrates that FRMD3 interacts with the actin and tubulin cytoskeleton at adherens junctions and that a reduction in FRMD3 levels leads to dysregulation of junctional components as well as an increase in proapoptotic activity in human kidney tubule cells. Taken together, this work suggests a novel role for FRMD3 in maintaining cell and junctional integrity within the kidney tubule.

## Supplementary Material

## Data Availability

Data related to transcriptomic, proteomic, or metabolomic data. Experimental Data. Raw Data/Source Data. Gene Expression Omnibus. PRoteomics IDEntifications database. https://www.ebi.ac.uk/pride/archive/projects/PXD015297/. https://www.ncbi.nlm.nih.gov/geo/query/acc.cgi?acc=GSE137570.

## Supplemental Material

GENIE Consortium—Full List of Members

Supplemental Methods

References

Supplemental Figure 1. Correlation of Clinical variables of kidney disease within the NDRBB cohorts.

Supplemental Figure 2. Enriched terms in genes correlated both positively and negatively with either eGFR or %TIF in patient biopsy samples.

Supplemental Figure 3. Gene set enrichment analysis highlights the overlapping processes between disease severity and future disease progression.

Supplemental Figure 4. FRMD3 expression in microarray analysis of microdissected kidney biopsy samples.

Supplemental Figure 5. FRMD3 is enriched at the periphery of HK-2 cells at high density.

Supplemental Figure 6. Full-length Western blot images used for densitometry in Figure 5.

Supplemental Figure 7. FRMD3 knockdown does not result in significant alterations in long-chain fatty acid oxidation in HK-2 kidney proximal tubule cells.

Supplemental Table 1. Histology.

Supplemental Table 2. Genes correlating with eGFR.

Supplemental Table 3. Genes correlating with %TIF.

Supplemental Table 4. Gene set enrichment for genes associated with eGFR.

Supplemental Table 5. Gene set enrichment for genes associated with %TIF.

Supplemental Table 6. Genes which show correlation with both eGFR and %TIF.

Supplemental Table 7. Gene set enrichment for gene expression associated with more severe disease (lower eGFR and higher %TIF).

Supplemental Table 8. Differentially expressed genes in the setting of progressive CKD compared with stable CKD.

Supplemental Table 9. Gene set enrichment for genes with greater expression in progressive CKD compared with stable CKD.

Supplemental Table 10. Genes which show correlation with both eGFR and %TIF and differential expression between progressive and Stable CKD.

Supplemental Table 11. Gene expression correlated with eGFR and fibrosis in microdissected tubule samples.

Supplemental Table 12. Top ranked kidney transcripts associated with CKD status, sorted by eGFR correlation in an external cohort of microdissected tubule samples.

Supplemental Table 13. Differentially expressed genes between pseudo-bulked CKD and healthy reference snRNA-seq samples within the KPMP all cells.

Supplemental Table 14. Differentially expressed genes between pseudo-bulked CKD and healthy reference snRNA-seq samples within the KPMP proximal tubule clusters.

Supplemental Table 15. FRMD3-V5 interactors by affinity purification mass spectrometry.

Supplemental Table 16. Ingenuity pathway analysis results for FRMD3 interactants.
