# Supplementary material for: A Novel Role for FERM Domain-Containing Protein 3 in CKD
Source: Kidney360. 2024 Oct 16;5(12):1799–812. doi: 10.34067/KID.0000000602 (PMC11687992; doi:10.34067/KID.0000000602)
Supplement: Supplementary file 1 [file kidney360-5-1799-s001.pdf]

ASN American Society of Nephrology | JASN Journal of the American Society of Nephrology | KIDNEY360 Accessing Our World From Every Angle | CJASN Clinical Journal of the American Society of Nephrology

# ASN Journal Disclosure Form

As per ASN journal policy, I have disclosed any financial relationships or commitments I have held in the past 36 months as included below. I have listed my Current Employer below to indicate there is a relationship requiring disclosure. If no relationship exists, my Current Employer is not listed.

D. Andrews reports the following:
Employer: University College Dublin

I understand that the information above will be published within the journal article, if accepted, and that failure to comply and/or to accurately and completely report the potential financial conflicts of interest could lead to the following: 1) Prior to publication, article rejection, or 2) Post-publication, sanctions ranging from, but not limited to, issuing a correction, reporting the inaccurate information to the authors' institution, banning authors from submitting work to ASN journals for varying lengths of time, and/or retraction of the published work.

Name: Darrell C. Andrews
Manuscript ID: K360-2024-000159R2
Manuscript Title: A novel role for FERM domain-containing protein 3 (FRMD3) in chronic kidney disease.
Date of Completion: August 19, 2024
Disclosure Updated Date: August 19, 2024

ASN
American Society of Nephrology
JASN
Journal of the American Society of Nephrology
KIDNEY360
Accessing Our World From Every Angle
CJASN
Clinical Journal of the American Society of Nephrology

# ASN Journal Disclosure Form

As per ASN journal policy, I have disclosed any financial relationships or commitments I have held in the past 36 months as included below. I have listed my Current Employer below to indicate there is a relationship requiring disclosure. If no relationship exists, my Current Employer is not listed.

E. Brennan reports the following:
Employer: University College Dublin; and Ownership Interest: Attenuate Therapeutics Ltd.

I understand that the information above will be published within the journal article, if accepted, and that failure to comply and/or to accurately and completely report the potential financial conflicts of interest could lead to the following: 1) Prior to publication, article rejection, or 2) Post-publication, sanctions ranging from, but not limited to, issuing a correction, reporting the inaccurate information to the authors' institution, banning authors from submitting work to ASN journals for varying lengths of time, and/or retraction of the published work.

Name: Eoin Brennan
Manuscript ID: K360-2024-000159R1
Manuscript Title: A novel role for FERM domain-containing protein 3 (FRMD3) in chronic kidney disease
Date of Completion: June 7, 2024
Disclosure Updated Date: June 7, 2024

ASN American Society of Nephrology JASN Journal of the American Society of Nephrology KIDNEY360 Accessing Our World From Every Angle CJASN Clinical Journal of the American Society of Nephrology

# ASN Journal Disclosure Form

As per ASN journal policy, I have disclosed any financial relationships or commitments I have held in the past 36 months as included below. I have listed my Current Employer below to indicate there is a relationship requiring disclosure. If no relationship exists, my Current Employer is not listed.

P. Conlon reports the following:
Employer: Beaumont Hospital, Dublin 9; and Research Funding: BioMarin Pharma.

I understand that the information above will be published within the journal article, if accepted, and that failure to comply and/or to accurately and completely report the potential financial conflicts of interest could lead to the following: 1) Prior to publication, article rejection, or 2) Post-publication, sanctions ranging from, but not limited to, issuing a correction, reporting the inaccurate information to the authors' institution, banning authors from submitting work to ASN journals for varying lengths of time, and/or retraction of the published work.

Name: Peter J. Conlon
Manuscript ID: k360-2024-000159R1
Manuscript Title: A novel form of FERM
Date of Completion: June 6, 2024
Disclosure Updated Date: May 19, 2024

ASN American Society of Nephrology JASN Journal of the American Society of Nephrology KIDNEY360 Accessing Our World From Every Angle CJASN Clinical Journal of the American Society of Nephrology

# ASN Journal Disclosure Form

As per ASN journal policy, I have disclosed any financial relationships or commitments I have held in the past 36 months as included below. I have listed my Current Employer below to indicate there is a relationship requiring disclosure. If no relationship exists, my Current Employer is not listed.

D. Crean reports the following:
Employer: University College Dublin, Ireland

I understand that the information above will be published within the journal article, if accepted, and that failure to comply and/or to accurately and completely report the potential financial conflicts of interest could lead to the following: 1) Prior to publication, article rejection, or 2) Post-publication, sanctions ranging from, but not limited to, issuing a correction, reporting the inaccurate information to the authors' institution, banning authors from submitting work to ASN journals for varying lengths of time, and/or retraction of the published work.

Name: Daniel Crean
Manuscript ID: K360-2024-000159R2
Manuscript Title: A novel role for FERM domain-containing protein 3 (FRMD3) in chronic kidney disease
Date of Completion: July 15, 2024
Disclosure Updated Date: July 15, 2024

ASN American Society of Nephrology | JASN Journal of the American Society of Nephrology | KIDNEY360 Accessing Our World From Every Angle | CJASN Clinical Journal of the American Society of Nephrology

# ASN Journal Disclosure Form

As per ASN journal policy, I have disclosed any financial relationships or commitments I have held in the past 36 months as included below. I have listed my Current Employer below to indicate there is a relationship requiring disclosure. If no relationship exists, my Current Employer is not listed.

B. Crifo reports the following:
Employer: University College Dublin

I understand that the information above will be published within the journal article, if accepted, and that failure to comply and/or to accurately and completely report the potential financial conflicts of interest could lead to the following: 1) Prior to publication, article rejection, or 2) Post-publication, sanctions ranging from, but not limited to, issuing a correction, reporting the inaccurate information to the authors' institution, banning authors from submitting work to ASN journals for varying lengths of time, and/or retraction of the published work.

Name: Bianca Crifo
Manuscript ID: K360-2024-000159R1
Manuscript Title: A novel role for FERM domain-containing protein 3 (FRMD3) in chronic kidney disease
Date of Completion: June 9, 2024
Disclosure Updated Date: June 9, 2024

ASN
American Society of Nephrology
JASN
Journal of the American Society of Nephrology
KIDNEY360
Accessing Our World From Every Angle
CJASN
Clinical Journal of the American Society of Nephrology

# ASN Journal Disclosure Form

As per ASN journal policy, I have disclosed any financial relationships or commitments I have held in the past 36 months as included below. I have listed my Current Employer below to indicate there is a relationship requiring disclosure. If no relationship exists, my Current Employer is not listed.

E. Dillon has nothing to disclose.

I understand that the information above will be published within the journal article, if accepted, and that failure to comply and/or to accurately and completely report the potential financial conflicts of interest could lead to the following: 1) Prior to publication, article rejection, or 2) Post-publication, sanctions ranging from, but not limited to, issuing a correction, reporting the inaccurate information to the authors' institution, banning authors from submitting work to ASN journals for varying lengths of time, and/or retraction of the published work.

Name: Eugene T Dillon
Manuscript ID: K360-2024-000159R2
Manuscript Title: A novel role for FERM domain-containing protein 3 (FRMD3) in chronic kidney disease.
Date of Completion: August 9, 2024
Disclosure Updated Date: August 9, 2024

ASN
American Society of Nephrology
JASN
Journal of the American Society of Nephrology
KIDNEY360
Accessing Our World From Every Angle
CJASN
Clinical Journal of the American Society of Nephrology

# ASN Journal Disclosure Form

As per ASN journal policy, I have disclosed any financial relationships or commitments I have held in the past 36 months as included below. I have listed my Current Employer below to indicate there is a relationship requiring disclosure. If no relationship exists, my Current Employer is not listed.

A. Dorman has nothing to disclose.

I understand that the information above will be published within the journal article, if accepted, and that failure to comply and/or to accurately and completely report the potential financial conflicts of interest could lead to the following: 1) Prior to publication, article rejection, or 2) Post-publication, sanctions ranging from, but not limited to, issuing a correction, reporting the inaccurate information to the authors' institution, banning authors from submitting work to ASN journals for varying lengths of time, and/or retraction of the published work.

Name: Anthony M. Dorman
Manuscript ID: K360-2024-000159R1
Manuscript Title: A novel role for FERM domain-containing protein 3 (FRMD3) in chronic kidney disease
Date of Completion: August 23, 2024
Disclosure Updated Date: August 23, 2024

ASN American Society of Nephrology
JASN Journal of the American Society of Nephrology
KIDNEY360 Accessing Our World From Every Angle
CJASN Clinical Journal of the American Society of Nephrology

# ASN Journal Disclosure Form

As per ASN journal policy, I have disclosed any financial relationships or commitments I have held in the past 36 months as included below. I have listed my Current Employer below to indicate there is a relationship requiring disclosure. If no relationship exists, my Current Employer is not listed.

R. Doyle reports the following:
Employer: Mater Misericordiae University Hospital

I understand that the information above will be published within the journal article, if accepted, and that failure to comply and/or to accurately and completely report the potential financial conflicts of interest could lead to the following: 1) Prior to publication, article rejection, or 2) Post-publication, sanctions ranging from, but not limited to, issuing a correction, reporting the inaccurate information to the authors' institution, banning authors from submitting work to ASN journals for varying lengths of time, and/or retraction of the published work.

Name: Ross Doyle
Manuscript ID: K360-2024-000159R1
Manuscript Title: A novel role for FERM domain-containing protein 3 (FRMD3) in chronic kidney disease
Date of Completion: June 8, 2024
Disclosure Updated Date: June 8, 2024

ASN American Society of Nephrology
JASN Journal of the American Society of Nephrology
KIDNEY360 Accessing Our World From Every Angle
CJASN Clinical Journal of the American Society of Nephrology

# ASN Journal Disclosure Form

As per ASN journal policy, I have disclosed any financial relationships or commitments I have held in the past 36 months as included below. I have listed my Current Employer below to indicate there is a relationship requiring disclosure. If no relationship exists, my Current Employer is not listed.

J. Florez reports the following:
Employer: Massachusetts General Hospital; Consultancy: AstraZeneca, Novo Nordisk; Research Funding: Novo Nordisk; Honoraria: Novo Nordisk, AstraZeneca; and Other Interests or Relationships: Doris Duke Foundation.

I understand that the information above will be published within the journal article, if accepted, and that failure to comply and/or to accurately and completely report the potential financial conflicts of interest could lead to the following: 1) Prior to publication, article rejection, or 2) Post-publication, sanctions ranging from, but not limited to, issuing a correction, reporting the inaccurate information to the authors' institution, banning authors from submitting work to ASN journals for varying lengths of time, and/or retraction of the published work.

Name: Jose C. Florez
Manuscript ID: K360-2024-000159R2
Manuscript Title: A novel role for FERM domain-containing protein 3 (FRMD3) in chronic kidney disease.
Date of Completion: August 30, 2024
Disclosure Updated Date: May 17, 2024

ASN American Society of Nephrology
JASN Journal of the American Society of Nephrology
KIDNEY360 Accessing Our World From Every Angle
CJASN Clinical Journal of the American Society of Nephrology

# ASN Journal Disclosure Form

As per ASN journal policy, I have disclosed any financial relationships or commitments I have held in the past 36 months as included below. I have listed my Current Employer below to indicate there is a relationship requiring disclosure. If no relationship exists, my Current Employer is not listed.

A. Gaffney has nothing to disclose.

I understand that the information above will be published within the journal article, if accepted, and that failure to comply and/or to accurately and completely report the potential financial conflicts of interest could lead to the following: 1) Prior to publication, article rejection, or 2) Post-publication, sanctions ranging from, but not limited to, issuing a correction, reporting the inaccurate information to the authors' institution, banning authors from submitting work to ASN journals for varying lengths of time, and/or retraction of the published work.

Name: Andrew Gaffney
Manuscript ID: K360-2024-000159R2
Manuscript Title: A novel role for FERM domain-containing protein 3 (FRMD3) in chronic kidney disease
Date of Completion: July 16, 2024
Disclosure Updated Date: July 16, 2024

ASN American Society of Nephrology | JASN Journal of the American Society of Nephrology | KIDNEY360 Accessing Our World From Every Angle | CJASN Clinical Journal of the American Society of Nephrology

# ASN Journal Disclosure Form

As per ASN journal policy, I have disclosed any financial relationships or commitments I have held in the past 36 months as included below. I have listed my Current Employer below to indicate there is a relationship requiring disclosure. If no relationship exists, my Current Employer is not listed.

C. Godson reports the following:
Employer: University College Dublin; Ownership Interest: Attenuate Therapeutics. Founder, shareholder, incorporated 2022; Spouse: Open Orphan plc.; and Advisory or Leadership Role: Trustee Bart's Charity, London; Board Member Irish Research Council., Science Secretary, Royal Irish Academy; ASBMB [Editorial Board J Biol Chem; Mol Pharm]; Br J Pharmacology [Editorial Board]. Non executive Director Kerry group plc.

I understand that the information above will be published within the journal article, if accepted, and that failure to comply and/or to accurately and completely report the potential financial conflicts of interest could lead to the following: 1) Prior to publication, article rejection, or 2) Post-publication, sanctions ranging from, but not limited to, issuing a correction, reporting the inaccurate information to the authors' institution, banning authors from submitting work to ASN journals for varying lengths of time, and/or retraction of the published work.

Name: Catherine Godson
Manuscript ID: K360-2024-000159R2
Manuscript Title: A novel role for FERM domain-containing protein 3 (FRMD3) in chronic kidney disease
Date of Completion: July 16, 2024
Disclosure Updated Date: May 24, 2024

ASN American Society of Nephrology JASN Journal of the American Society of Nephrology KIDNEY360 Accessing Our World From Every Angle CJASN Clinical Journal of the American Society of Nephrology

# ASN Journal Disclosure Form

As per ASN journal policy, I have disclosed any financial relationships or commitments I have held in the past 36 months as included below. I have listed my Current Employer below to indicate there is a relationship requiring disclosure. If no relationship exists, my Current Employer is not listed.

O. Gough reports the following:
Employer: University College Dublin - National University of Ireland: University College Dublin; AbbVie Ltd. (Ireland); and Ownership Interest: Abbvie Inc. (NYSE: ABBV).

I understand that the information above will be published within the journal article, if accepted, and that failure to comply and/or to accurately and completely report the potential financial conflicts of interest could lead to the following: 1) Prior to publication, article rejection, or 2) Post-publication, sanctions ranging from, but not limited to, issuing a correction, reporting the inaccurate information to the authors' institution, banning authors from submitting work to ASN journals for varying lengths of time, and/or retraction of the published work.

Name: Oisín Shane Gough
Manuscript ID: K360-2024-000159R1
Manuscript Title: A novel role for FERM domain-containing protein 3 (FRMD3) in chronic kidney disease.
Date of Completion: June 5, 2024
Disclosure Updated Date: June 5, 2024

ASN American Society of Nephrology | JASN Journal of the American Society of Nephrology | KIDNEY360 Accessing Our World From Every Angle | CJASN Clinical Journal of the American Society of Nephrology

# ASN Journal Disclosure Form

As per ASN journal policy, I have disclosed any financial relationships or commitments I have held in the past 36 months as included below. I have listed my Current Employer below to indicate there is a relationship requiring disclosure. If no relationship exists, my Current Employer is not listed.

P. Groop reports the following:
Employer: University of Helsinki and Helsinki University Hospital; Folkhälsan Research Center; Research Funding: Eli Lilly, Roche (>10 years ago); Honoraria: Lecture fees from Astellas, Astra Zeneca, Bayer, Boehringer Ingelheim, Eli Lilly, Genzyme, Medscape, MSD, Mundipharma, Novartis, Novo Nordisk, PeerVoice, Sanofi and SCIARC; Advisory or Leadership Role: Member of Advisory Boards for Astellas, AbbVie, Astra Zeneca, Bayer, Boehringer Ingelheim, Cebix, Eli Lilly, Janssen, Medscape, MSD, Mundipharma, Nestlé, Novartis, Novo Nordisk and Sanofi; Speakers Bureau: Lecture fees from Astellas, Astra Zeneca, Bayer, Boehringer Ingelheim, Eli Lilly, Genzyme, Medscape, MSD, Mundipharma, Novartis, Novo Nordisk, PeerVoice, Sanofi and SCIARC; and Other Interests or Relationships: Chairman of the Board of the Signe and Ane Gyllenberg Foundation.

I understand that the information above will be published within the journal article, if accepted, and that failure to comply and/or to accurately and completely report the potential financial conflicts of interest could lead to the following: 1) Prior to publication, article rejection, or 2) Post-publication, sanctions ranging from, but not limited to, issuing a correction, reporting the inaccurate information to the authors' institution, banning authors from submitting work to ASN journals for varying lengths of time, and/or retraction of the published work.

Name: Per-Henrik Groop
Manuscript ID: K360-2024-000159R2
Manuscript Title: A novel role for FERM domain-containing protein 3 (FRMD3) in chronic kidney disease
Date of Completion: August 21, 2024
Disclosure Updated Date: August 21, 2024

ASN American Society of Nephrology | JASN Journal of the American Society of Nephrology | KIDNEY360 Accessing Our World From Every Angle | CJASN Clinical Journal of the American Society of Nephrology

# ASN Journal Disclosure Form

As per ASN journal policy, I have disclosed any financial relationships or commitments I have held in the past 36 months as included below. I have listed my Current Employer below to indicate there is a relationship requiring disclosure. If no relationship exists, my Current Employer is not listed.

J. Hirschhorn reports the following:
Employer: Boston Children's Hospital; Ownership Interest: Camp4 Therapeutics; and Research Funding: Pfizer.

I understand that the information above will be published within the journal article, if accepted, and that failure to comply and/or to accurately and completely report the potential financial conflicts of interest could lead to the following: 1) Prior to publication, article rejection, or 2) Post-publication, sanctions ranging from, but not limited to, issuing a correction, reporting the inaccurate information to the authors' institution, banning authors from submitting work to ASN journals for varying lengths of time, and/or retraction of the published work.

Name: Joel Hirschhorn
Manuscript ID: K360-2024-000159R2
Manuscript Title: A novel role for FERM domain-containing protein 3 (FRMD3) in chronic kidney disease
Date of Completion: August 21, 2024
Disclosure Updated Date: August 21, 2024

ASN American Society of Nephrology
JASN Journal of the American Society of Nephrology
KIDNEY360 Accessing Our World From Every Angle
CJASN Clinical Journal of the American Society of Nephrology

# ASN Journal Disclosure Form

As per ASN journal policy, I have disclosed any financial relationships or commitments I have held in the past 36 months as included below. I have listed my Current Employer below to indicate there is a relationship requiring disclosure. If no relationship exists, my Current Employer is not listed.

M. Hughes reports the following:
Employer: University College Dublin; Novartis Pharmaceuticals (Ireland plc) (since May 2019), in R&D, cardiovascular disease (not kidney disease);; and Ownership Interest: Novartis Pharmaceuticals, Global Business Solutions, Novartis Corporate Center, Dublin, Ireland. ;.

I understand that the information above will be published within the journal article, if accepted, and that failure to comply and/or to accurately and completely report the potential financial conflicts of interest could lead to the following: 1) Prior to publication, article rejection, or 2) Post-publication, sanctions ranging from, but not limited to, issuing a correction, reporting the inaccurate information to the authors' institution, banning authors from submitting work to ASN journals for varying lengths of time, and/or retraction of the published work.

Name: Maria F Hughes
Manuscript ID: K360-2024-000159R2
Manuscript Title: A novel role for FERM domain-containing protein 3 (FRMD3) in chronic kidney disease
Date of Completion: August 9, 2024
Disclosure Updated Date: August 9, 2024

ASN American Society of Nephrology | JASN Journal of the American Society of Nephrology | KIDNEY360 Accessing Our World From Every Angle | CJASN Clinical Journal of the American Society of Nephrology

# ASN Journal Disclosure Form

As per ASN journal policy, I have disclosed any financial relationships or commitments I have held in the past 36 months as included below. I have listed my Current Employer below to indicate there is a relationship requiring disclosure. If no relationship exists, my Current Employer is not listed.

C. Kennedy reports the following:
Employer: University College Dublin

I understand that the information above will be published within the journal article, if accepted, and that failure to comply and/or to accurately and completely report the potential financial conflicts of interest could lead to the following: 1) Prior to publication, article rejection, or 2) Post-publication, sanctions ranging from, but not limited to, issuing a correction, reporting the inaccurate information to the authors' institution, banning authors from submitting work to ASN journals for varying lengths of time, and/or retraction of the published work.

Name: Ciarán Kennedy
Manuscript ID: K360-2024-000159R1
Manuscript Title: A novel role for FERM domain-containing protein 3 (FRMD3) in chronic kidney disease.
Date of Completion: June 10, 2024
Disclosure Updated Date: June 10, 2024

ASN American Society of Nephrology
JASN Journal of the American Society of Nephrology
KIDNEY360 Accessing Our World From Every Angle
CJASN Clinical Journal of the American Society of Nephrology

# ASN Journal Disclosure Form

As per ASN journal policy, I have disclosed any financial relationships or commitments I have held in the past 36 months as included below. I have listed my Current Employer below to indicate there is a relationship requiring disclosure. If no relationship exists, my Current Employer is not listed.

S. Kennedy reports the following:
Employer: TriviumVet

I understand that the information above will be published within the journal article, if accepted, and that failure to comply and/or to accurately and completely report the potential financial conflicts of interest could lead to the following: 1) Prior to publication, article rejection, or 2) Post-publication, sanctions ranging from, but not limited to, issuing a correction, reporting the inaccurate information to the authors' institution, banning authors from submitting work to ASN journals for varying lengths of time, and/or retraction of the published work.

Name: Susan A Kennedy
Manuscript ID: K360-2024-000159R2
Manuscript Title: A novel role for FERM domain-containing protein 3 (FRMD3) in chronic kidney disease.
Date of Completion: July 15, 2024
Disclosure Updated Date: July 15, 2024

ASN American Society of Nephrology | JASN Journal of the American Society of Nephrology | KIDNEY360 Accessing Our World From Every Angle | CJASN Clinical Journal of the American Society of Nephrology

# ASN Journal Disclosure Form

As per ASN journal policy, I have disclosed any financial relationships or commitments I have held in the past 36 months as included below. I have listed my Current Employer below to indicate there is a relationship requiring disclosure. If no relationship exists, my Current Employer is not listed.

M. Kretzler reports the following:
Employer: University of Michigan Medical School; Consultancy: None by me personally.; As employee of U Michigan for: Janssen, NovoNordisk, Otsuka, Alexion/Astra-Zeneca, Variant Bio, Novartis.; Research Funding: Sponsored research project as PI at U Michigan: National Institutes of Health, Chan Zuckerberg Initiative, JDRF, Alport Foundation, amfAR, AstraZeneca, NovoNordisk, Eli Lilly, Gilead, Janssen, Boehringer-Ingelheim, Moderna, European Union Innovative Medicine Initiative, Certa, Chinook, Angion, RenalytixAI, Travere, Regeneron, IONIS, Maze Therapeutics, Roche-Genentech, Sanofi, Dimerix.; Patents or Royalties: PCT/EP2014/073413 ?Biomarkers and methods for progression prediction for chronic kidney disease?; and Advisory or Leadership Role: Editorial Board:  J Am Soc Nephrology, Kidney International, Kidney Disease; Advisory Board Chair: Nephcure.

I understand that the information above will be published within the journal article, if accepted, and that failure to comply and/or to accurately and completely report the potential financial conflicts of interest could lead to the following: 1) Prior to publication, article rejection, or 2) Post-publication, sanctions ranging from, but not limited to, issuing a correction, reporting the inaccurate information to the authors' institution, banning authors from submitting work to ASN journals for varying lengths of time, and/or retraction of the published work.

Name: Matthias Kretzler
Manuscript ID:  K360-2024-000159R2
Manuscript Title: A novel role for FERM domain-containing protein 3 (FRMD3) in chronic kidney disease.
Date of Completion: August 20, 2024
Disclosure Updated Date: August 20, 2024

ASN American Society of Nephrology | JASN Journal of the American Society of Nephrology | KIDNEY360 Accessing Our World From Every Angle | CJASN Clinical Journal of the American Society of Nephrology

# ASN Journal Disclosure Form

As per ASN journal policy, I have disclosed any financial relationships or commitments I have held in the past 36 months as included below. I have listed my Current Employer below to indicate there is a relationship requiring disclosure. If no relationship exists, my Current Employer is not listed.

F. Martin has nothing to disclose.

I understand that the information above will be published within the journal article, if accepted, and that failure to comply and/or to accurately and completely report the potential financial conflicts of interest could lead to the following: 1) Prior to publication, article rejection, or 2) Post-publication, sanctions ranging from, but not limited to, issuing a correction, reporting the inaccurate information to the authors' institution, banning authors from submitting work to ASN journals for varying lengths of time, and/or retraction of the published work.

Name: Finian Martin
Manuscript ID: K360-2024-000159R2
Manuscript Title: A novel role for FERM domain containing protein 3 (FRMD3) in chronic kidney disease
Date of Completion: August 21, 2024
Disclosure Updated Date: August 21, 2024

ASN American Society of Nephrology | JASN Journal of the American Society of Nephrology | KIDNEY360 Accessing Our World From Every Angle | CJASN Clinical Journal of the American Society of Nephrology

# ASN Journal Disclosure Form

As per ASN journal policy, I have disclosed any financial relationships or commitments I have held in the past 36 months as included below. I have listed my Current Employer below to indicate there is a relationship requiring disclosure. If no relationship exists, my Current Employer is not listed.

D. Matallanas reports the following:
Employer: University College Dublin; and Consultancy: Boehringer Ingelheim.

I understand that the information above will be published within the journal article, if accepted, and that failure to comply and/or to accurately and completely report the potential financial conflicts of interest could lead to the following: 1) Prior to publication, article rejection, or 2) Post-publication, sanctions ranging from, but not limited to, issuing a correction, reporting the inaccurate information to the authors' institution, banning authors from submitting work to ASN journals for varying lengths of time, and/or retraction of the published work.

Name: David Matallanas
Manuscript ID: K360-2024-000159R1
Manuscript Title: A novel role for FERM domain-containing protein 3 (FRMD3) in chronic kidney disease.,
Date of Completion: June 6, 2024
Disclosure Updated Date: June 6, 2024

ASN American Society of Nephrology JASN Journal of the American Society of Nephrology KIDNEY360 Accessing Our World From Every Angle CJASN Clinical Journal of the American Society of Nephrology

# ASN Journal Disclosure Form

As per ASN journal policy, I have disclosed any financial relationships or commitments I have held in the past 36 months as included below. I have listed my Current Employer below to indicate there is a relationship requiring disclosure. If no relationship exists, my Current Employer is not listed.

A. Maxwell has nothing to disclose.

I understand that the information above will be published within the journal article, if accepted, and that failure to comply and/or to accurately and completely report the potential financial conflicts of interest could lead to the following: 1) Prior to publication, article rejection, or 2) Post-publication, sanctions ranging from, but not limited to, issuing a correction, reporting the inaccurate information to the authors' institution, banning authors from submitting work to ASN journals for varying lengths of time, and/or retraction of the published work.

Name: Alexander P. Maxwell
Manuscript ID: K360-2024-000159R1
Manuscript Title: "A novel role for FERM domain-containing protein 3 (FRMD3) in chronic kidney disease.,"
Date of Completion: June 6, 2024
Disclosure Updated Date: June 6, 2024

ASN American Society of Nephrology | JASN Journal of the American Society of Nephrology | KIDNEY360 Accessing Our World From Every Angle | CJASN Clinical Journal of the American Society of Nephrology

# ASN Journal Disclosure Form

As per ASN journal policy, I have disclosed any financial relationships or commitments I have held in the past 36 months as included below. I have listed my Current Employer below to indicate there is a relationship requiring disclosure. If no relationship exists, my Current Employer is not listed.

S. McAnallen reports the following:
Employer: St. James's Hospital, Dublin, Ireland; Beaumont Hospital, Dublin Ireland

I understand that the information above will be published within the journal article, if accepted, and that failure to comply and/or to accurately and completely report the potential financial conflicts of interest could lead to the following: 1) Prior to publication, article rejection, or 2) Post-publication, sanctions ranging from, but not limited to, issuing a correction, reporting the inaccurate information to the authors' institution, banning authors from submitting work to ASN journals for varying lengths of time, and/or retraction of the published work.

Name: Susan Marie McAnallen
Manuscript ID: K360-2024-000159R1
Manuscript Title: A novel role for FERM domain-containing protein 3 (FRMD3) in chronic kidney disease
Date of Completion: June 4, 2024
Disclosure Updated Date: May 22, 2024

ASN American Society of Nephrology
JASN Journal of the American Society of Nephrology
KIDNEY360 Accessing Our World From Every Angle
CJASN Clinical Journal of the American Society of Nephrology

# ASN Journal Disclosure Form

As per ASN journal policy, I have disclosed any financial relationships or commitments I have held in the past 36 months as included below. I have listed my Current Employer below to indicate there is a relationship requiring disclosure. If no relationship exists, my Current Employer is not listed.

C. McEvoy reports the following:
Employer: Tallaght University Hospital, Dublin

I understand that the information above will be published within the journal article, if accepted, and that failure to comply and/or to accurately and completely report the potential financial conflicts of interest could lead to the following: 1) Prior to publication, article rejection, or 2) Post-publication, sanctions ranging from, but not limited to, issuing a correction, reporting the inaccurate information to the authors' institution, banning authors from submitting work to ASN journals for varying lengths of time, and/or retraction of the published work.

Name: Caitríona M. McEvoy
Manuscript ID: K360-2024-000159R2
Manuscript Title: A novel role for FERM domain-containing protein 3 (FRMD3) in chronic kidney disease
Date of Completion: August 13, 2024
Disclosure Updated Date: August 13, 2024

ASN American Society of Nephrology JASN Journal of the American Society of Nephrology KIDNEY360 Accessing Our World From Every Angle CJASN Clinical Journal of the American Society of Nephrology

# ASN Journal Disclosure Form

As per ASN journal policy, I have disclosed any financial relationships or commitments I have held in the past 36 months as included below. I have listed my Current Employer below to indicate there is a relationship requiring disclosure. If no relationship exists, my Current Employer is not listed.

G. McKay has nothing to disclose.

I understand that the information above will be published within the journal article, if accepted, and that failure to comply and/or to accurately and completely report the potential financial conflicts of interest could lead to the following: 1) Prior to publication, article rejection, or 2) Post-publication, sanctions ranging from, but not limited to, issuing a correction, reporting the inaccurate information to the authors' institution, banning authors from submitting work to ASN journals for varying lengths of time, and/or retraction of the published work.

Name: Gareth J. McKay
Manuscript ID: K360-2024-000159R1
Manuscript Title: A novel role for FERM domain-containing protein 3 (FRMD3) in chronic kidney disease
Date of Completion: June 5, 2024
Disclosure Updated Date: May 19, 2024

ASN
American Society of Nephrology
JASN
Journal of the American Society of Nephrology
KIDNEY360
Accessing Our World From Every Angle
CJASN
Clinical Journal of the American Society of Nephrology

# ASN Journal Disclosure Form

As per ASN journal policy, I have disclosed any financial relationships or commitments I have held in the past 36 months as included below. I have listed my Current Employer below to indicate there is a relationship requiring disclosure. If no relationship exists, my Current Employer is not listed.

A. Mcknight reports the following:
Employer: Queen's University of Belfast; Consultancy: Alnylam Pharmaceuticals; Research Funding: Multiomic Health Ltd; and Advisory or Leadership Role: Member of editorial board of BMC Nephrology and Genes, grant committees, and advisory boards for UK parliament, NI Department of Health, and Royal Irish Academy - none paid.

I understand that the information above will be published within the journal article, if accepted, and that failure to comply and/or to accurately and completely report the potential financial conflicts of interest could lead to the following: 1) Prior to publication, article rejection, or 2) Post-publication, sanctions ranging from, but not limited to, issuing a correction, reporting the inaccurate information to the authors' institution, banning authors from submitting work to ASN journals for varying lengths of time, and/or retraction of the published work.

Name: A.J. Mcknight
Manuscript ID: K360-2024-000159R2
Manuscript Title: A novel role for FERM domain-containing protein 3 (FRMD3) in chronic kidney disease
Date of Completion: August 13, 2024
Disclosure Updated Date: August 8, 2024

ASN American Society of Nephrology | JASN Journal of the American Society of Nephrology | KIDNEY360 Accessing Our World From Every Angle | CJASN Clinical Journal of the American Society of Nephrology

# ASN Journal Disclosure Form

As per ASN journal policy, I have disclosed any financial relationships or commitments I have held in the past 36 months as included below. I have listed my Current Employer below to indicate there is a relationship requiring disclosure. If no relationship exists, my Current Employer is not listed.

V. Nair has nothing to disclose.

I understand that the information above will be published within the journal article, if accepted, and that failure to comply and/or to accurately and completely report the potential financial conflicts of interest could lead to the following: 1) Prior to publication, article rejection, or 2) Post-publication, sanctions ranging from, but not limited to, issuing a correction, reporting the inaccurate information to the authors' institution, banning authors from submitting work to ASN journals for varying lengths of time, and/or retraction of the published work.

Name: Viji Nair
Manuscript ID: K360-2024-000159R2
Manuscript Title: A novel role for FERM domain-containing protein 3 (FRMD3) in chronic kidney disease
Date of Completion: July 15, 2024
Disclosure Updated Date: May 8, 2024

ASN American Society of Nephrology | JASN Journal of the American Society of Nephrology | KIDNEY360 Accessing Our World From Every Angle | CJASN Clinical Journal of the American Society of Nephrology

# ASN Journal Disclosure Form

As per ASN journal policy, I have disclosed any financial relationships or commitments I have held in the past 36 months as included below. I have listed my Current Employer below to indicate there is a relationship requiring disclosure. If no relationship exists, my Current Employer is not listed.

J. Rodriguez Martinez reports the following:
Employer: csic-ibbtec

I understand that the information above will be published within the journal article, if accepted, and that failure to comply and/or to accurately and completely report the potential financial conflicts of interest could lead to the following: 1) Prior to publication, article rejection, or 2) Post-publication, sanctions ranging from, but not limited to, issuing a correction, reporting the inaccurate information to the authors' institution, banning authors from submitting work to ASN journals for varying lengths of time, and/or retraction of the published work.

Name: Javier Rodriguez Martinez
Manuscript ID: K360-2024-000159R1
Manuscript Title: A novel role for FERM domain-containing protein 3 (FRMD3) in chronic kidney disease.
Date of Completion: June 6, 2024
Disclosure Updated Date: June 6, 2024

ASN American Society of Nephrology | JASN Journal of the American Society of Nephrology | KIDNEY360 Accessing Our World From Every Angle | CJASN Clinical Journal of the American Society of Nephrology

# ASN Journal Disclosure Form

As per ASN journal policy, I have disclosed any financial relationships or commitments I have held in the past 36 months as included below. I have listed my Current Employer below to indicate there is a relationship requiring disclosure. If no relationship exists, my Current Employer is not listed.

D. Sadlier reports the following:
Employer: Mater Misericordiae University Hospital, Dublin, Ireland; and Honoraria: Sanofi 2022.

I understand that the information above will be published within the journal article, if accepted, and that failure to comply and/or to accurately and completely report the potential financial conflicts of interest could lead to the following: 1) Prior to publication, article rejection, or 2) Post-publication, sanctions ranging from, but not limited to, issuing a correction, reporting the inaccurate information to the authors' institution, banning authors from submitting work to ASN journals for varying lengths of time, and/or retraction of the published work.

Name: Denise M. Sadlier
Manuscript ID: K360-2024-000159R2
Manuscript Title: A novel role for FERM domain-containing protein 3 (FRMD3) in chronic kidney disease
Date of Completion: August 28, 2024
Disclosure Updated Date: May 22, 2024

ASN American Society of Nephrology | JASN Journal of the American Society of Nephrology | KIDNEY360 Accessing Our World From Every Angle | CJASN Clinical Journal of the American Society of Nephrology

# ASN Journal Disclosure Form

As per ASN journal policy, I have disclosed any financial relationships or commitments I have held in the past 36 months as included below. I have listed my Current Employer below to indicate there is a relationship requiring disclosure. If no relationship exists, my Current Employer is not listed.

N. Sandholm reports the following:
Employer: Folkhälsan Research Center

I understand that the information above will be published within the journal article, if accepted, and that failure to comply and/or to accurately and completely report the potential financial conflicts of interest could lead to the following: 1) Prior to publication, article rejection, or 2) Post-publication, sanctions ranging from, but not limited to, issuing a correction, reporting the inaccurate information to the authors' institution, banning authors from submitting work to ASN journals for varying lengths of time, and/or retraction of the published work.

Name: Niina Sandholm
Manuscript ID: K360-2024-000159R1
Manuscript Title: A novel role for FERM domain-containing protein 3 (FRMD3) in chronic kidney disease.
Date of Completion: June 7, 2024
Disclosure Updated Date: May 20, 2024

ASN American Society of Nephrology JASN Journal of the American Society of Nephrology KIDNEY360 Accessing Our World From Every Angle CJASN Clinical Journal of the American Society of Nephrology

# ASN Journal Disclosure Form

As per ASN journal policy, I have disclosed any financial relationships or commitments I have held in the past 36 months as included below. I have listed my Current Employer below to indicate there is a relationship requiring disclosure. If no relationship exists, my Current Employer is not listed.

X. Sheng reports the following:
Employer: Zhejiang University

I understand that the information above will be published within the journal article, if accepted, and that failure to comply and/or to accurately and completely report the potential financial conflicts of interest could lead to the following: 1) Prior to publication, article rejection, or 2) Post-publication, sanctions ranging from, but not limited to, issuing a correction, reporting the inaccurate information to the authors' institution, banning authors from submitting work to ASN journals for varying lengths of time, and/or retraction of the published work.

Name: Xin Sheng
Manuscript ID: K360-2024-000159R1
Manuscript Title: A novel role for FERM domain-containing protein 3 (FRMD3) in chronic kidney disease
Date of Completion: June 7, 2024
Disclosure Updated Date: June 7, 2024

ASN American Society of Nephrology | JASN Journal of the American Society of Nephrology | KIDNEY360 Accessing Our World From Every Angle | CJASN Clinical Journal of the American Society of Nephrology

# ASN Journal Disclosure Form

As per ASN journal policy, I have disclosed any financial relationships or commitments I have held in the past 36 months as included below. I have listed my Current Employer below to indicate there is a relationship requiring disclosure. If no relationship exists, my Current Employer is not listed.

K. Susztak reports the following:
Employer: University of Pennsylvania, Perelman School of Medicine; Consultancy: Astra Zeneca, Pfizer, Janna, Otsuka, Novo Nordisk; Ownership Interest: Jnana, TarnaTx; Research Funding: Boehringer Ingelheim; Gilead; Regeneron; GSK, Novo Nordisk, Maze, Calico, Novartis,  Astra Zeneca, Kyowa Kirin Genentech, Variant Bio, ONO Pharma; Honoraria: Maze, Jnana, Otsuka, Pfizer; and Advisory or Leadership Role: Editorial board; Kidney International, Journal of Clinical Investigation, Cell Metabolism, Med, eBioMedicine, Journal of American Society of Nephrology, Jnana, Pfizer, Otsuka.

I understand that the information above will be published within the journal article, if accepted, and that failure to comply and/or to accurately and completely report the potential financial conflicts of interest could lead to the following: 1) Prior to publication, article rejection, or 2) Post-publication, sanctions ranging from, but not limited to, issuing a correction, reporting the inaccurate information to the authors' institution, banning authors from submitting work to ASN journals for varying lengths of time, and/or retraction of the published work.

Name: Katalin Susztak
Manuscript ID: K360-2024-000159R1
Manuscript Title: A novel role for FERM domain-containing protein 3 (FRMD3) in chronic kidney disease
Date of Completion: June 8, 2024
Disclosure Updated Date: May 28, 2024

ASN American Society of Nephrology
JASN Journal of the American Society of Nephrology
KIDNEY360 Accessing Our World From Every Angle
CJASN Clinical Journal of the American Society of Nephrology

# ASN Journal Disclosure Form

As per ASN journal policy, I have disclosed any financial relationships or commitments I have held in the past 36 months as included below. I have listed my Current Employer below to indicate there is a relationship requiring disclosure. If no relationship exists, my Current Employer is not listed.

Z. Yi reports the following:
Employer: Icahn School of Medicine at Mount Sinai

I understand that the information above will be published within the journal article, if accepted, and that failure to comply and/or to accurately and completely report the potential financial conflicts of interest could lead to the following: 1) Prior to publication, article rejection, or 2) Post-publication, sanctions ranging from, but not limited to, issuing a correction, reporting the inaccurate information to the authors' institution, banning authors from submitting work to ASN journals for varying lengths of time, and/or retraction of the published work.

Name: Zhengzi Yi
Manuscript ID: K360-2024-000159R1
Manuscript Title: A novel role for FERM domain-containing protein 3 (FRMD3) in chronic kidney disease.
Date of Completion: June 6, 2024
Disclosure Updated Date: May 21, 2024

ASN American Society of Nephrology JASN Journal of the American Society of Nephrology KIDNEY360 Accessing Our World From Every Angle CJASN Clinical Journal of the American Society of Nephrology

# ASN Journal Disclosure Form

As per ASN journal policy, I have disclosed any financial relationships or commitments I have held in the past 36 months as included below. I have listed my Current Employer below to indicate there is a relationship requiring disclosure. If no relationship exists, my Current Employer is not listed.

W. Zhang reports the following:
Consultancy: VericiDx; Patents or Royalties: 1. ref 27527-0134P01, Serial No. 61/951,651, filled March 2014. Method for identifying kidney allograft recipients at risk for chronic injury; 2. US Provisional Patent Application: Methods for Diagnosing Risk of Renal Allograft Fibrosis and Rejection (miRNA); 3. US Provisional Patent Application: Method For Diagnosing Subclinical Acute Rejection by RNA sequencing Analysis of A Predictive Gene Set; 4. US Provisional Patent Application: Pretransplant; and Advisory or Leadership Role: VericiDx.

I understand that the information above will be published within the journal article, if accepted, and that failure to comply and/or to accurately and completely report the potential financial conflicts of interest could lead to the following: 1) Prior to publication, article rejection, or 2) Post-publication, sanctions ranging from, but not limited to, issuing a correction, reporting the inaccurate information to the authors' institution, banning authors from submitting work to ASN journals for varying lengths of time, and/or retraction of the published work.

Name: Weijia Zhang
Manuscript ID: K360-2024-000159R2
Manuscript Title: A novel role for FERM domain-containing protein 3 (FRMD3) in chronic kidney disease
Date of Completion: August 21, 2024
Disclosure Updated Date: May 21, 2024
