# Supplementary material for: A Novel Role for FERM Domain-Containing Protein 3 in CKD
Source: Kidney360. 2024 Oct 16;5(12):1799–812. doi: 10.34067/KID.0000000602 (PMC11687992; doi:10.34067/KID.0000000602)
Supplement: Supplementary file 2 [file kidney360-5-1799-s002.pdf]

SUPPLEMENTAL MATERIAL

Contents

GENIE Consortium – FULL LIST OF MEMBERS 2
Supplemental Methods 5
Participant recruitment and Biobank 5
Human Renal Biopsies 5
RNA extraction, quantification, and quality assessment 5
RNA sequencing 6
Correlation Analysis 6
Differential Expression Analysis 6
Pathway Analysis 6
Analysis of publicly available Single-cell datasets. 7
FRMD3 in vitro studies 7
HK-2 cell culture 7
Lentiviral Particle Production 7
qRT-PCR 8
Immunofluorescence imaging of FRMD3-V5 expressing HK-2 cells. 8
Western Blot 8
Proteomic Analysis 9
Mitochondrial Function/Caspase Assays 9
Primary antibodies used in this study: 10
References 10
Supplemental Figure Legends: 11
S1: Correlation of Clinical variables of kidney disease within the NDRBB cohorts. 11
S2: Enriched terms in genes correlated both positively and negatively with either eGFR or %TIF in batient biopsy samples 11
S3: Gene set enrichment analysis highlights the overlapping processes between disease severity and future disease progression. 11
S4: FRMD3 expression in microarray analysis of microdiscected kidney biopsy samples. 12
S5: FRMD3 is enriched at the periphery of HK-2 cells at high density. 12
S6: Full length western blot images used for densitometry in Figure 5. 12
Supplemental Figures: 13

GENIE Consortium – FULL LIST OF MEMBERS

Name	Affiliations
**Massachusetts General Hospital and Broad Institute, Boston, MA, USA**	
Jose C Florez	Programs in Metabolism and Medical & Population Genetics, Broad Institute, Cambridge, MA USA<br>Diabetes Unit and Center for Genomic Medicine, Massachusetts General Hospital, Boston, MA USA.<br>Department of Medicine, Harvard Medical School, Boston, MA USA.
Joel N Hirschhorn	Programs in Metabolism and Medical & Population Genetics, Broad Institute, Cambridge, MA USA.<br>Division of Endocrinology, Boston Children's Hospital, Boston, MA, USA<br>Department of Pediatrics and Genetics, Harvard Medical School, Boston, MA, USA
Joanne B Cole	Department of Biomedical Informatics, University of Colorado School of Medicine.<br>Programs in Metabolism and Medical & Population Genetics, Broad Institute, Cambridge, MA USA.<br>Diabetes Unit and Center for Genomic Medicine, Massachusetts General Hospital, Boston, MA USA.
Raymond Kreienkamp	Division of Endocrinology, Boston Children's Hospital, Boston, MA, USA Diabetes Unit and Center for Genomic Medicine, Massachusetts General Hospital, Boston, MA USA.
**Queen's University Belfast, Belfast, Northern Ireland**	
Laura J Smyth	Molecular Epidemiology Research Group, Centre for Public Health, Queen's University Belfast, Belfast, UK.
Katie Kerr	Molecular Epidemiology Research Group, Centre for Public Health, Queen's University Belfast, Belfast, UK.
Jill Kilner	Molecular Epidemiology Research Group, Centre for Public Health, Queen's University Belfast, Belfast, UK.
Yogesh Gupta	Molecular Epidemiology Research Group, Centre for Public Health, Queen's University Belfast, Belfast, UK.
Claire Hill	Molecular Epidemiology Research Group, Centre for Public Health, Queen's University Belfast, Belfast, UK.
Christopher Wooster	Molecular Epidemiology Research Group, Centre for Public Health, Queen's University Belfast, Belfast, UK.
Kerry Anderson	Molecular Epidemiology Research Group, Centre for Public Health, Queen's University Belfast, Belfast, UK.
Gareth J McKay	Molecular Epidemiology Research Group, Centre for Public Health, Queen's University Belfast, Belfast, UK.
Amy Jayne McKnight	Molecular Epidemiology Research Group, Centre for Public Health, Queen's University Belfast, Belfast, UK.

Alexander P Maxwell	Molecular Epidemiology Research Group, Centre for Public Health, Queen's University Belfast, Belfast, UK.<br>Regional Nephrology Unit, Belfast City Hospital, Belfast, Northern Ireland, UK.
**The FinnDiane Study Group, Folkhälsan Research Center, Helsinki, Finland**	
Emma H Dahlström	Folkhälsan Institute of Genetics, Folkhälsan Research Center, Helsinki, Finland.<br>Department of Nephrology, University of Helsinki and Helsinki University Hospital, Helsinki, Finland.<br>Research Program for Clinical and Molecular Metabolism, Faculty of Medicine, University of Helsinki, 00290, Helsinki, Finland.
Anna Syreeni	Folkhälsan Institute of Genetics, Folkhälsan Research Center, Helsinki, Finland.<br>Department of Nephrology, University of Helsinki and Helsinki University Hospital, Helsinki, Finland.<br>Research Program for Clinical and Molecular Metabolism, Faculty of Medicine, University of Helsinki, 00290, Helsinki, Finland.
Erkka Valo	Folkhälsan Institute of Genetics, Folkhälsan Research Center, Helsinki, Finland.<br>Department of Nephrology, University of Helsinki and Helsinki University Hospital, Helsinki, Finland.<br>Research Program for Clinical and Molecular Metabolism, Faculty of Medicine, University of Helsinki, 00290, Helsinki, Finland.
Carol Forsblom	Folkhälsan Institute of Genetics, Folkhälsan Research Center, Helsinki, Finland.<br>Department of Nephrology, University of Helsinki and Helsinki University Hospital, Helsinki, Finland.<br>Research Program for Clinical and Molecular Metabolism, Faculty of Medicine, University of Helsinki, 00290, Helsinki, Finland.<br>Deceased.
Niina Sandholm	Folkhälsan Institute of Genetics, Folkhälsan Research Center, Helsinki, Finland.<br>Department of Nephrology, University of Helsinki and Helsinki University Hospital, Helsinki, Finland.<br>Research Program for Clinical and Molecular Metabolism, Faculty of Medicine, University of Helsinki, 00290, Helsinki, Finland.
Per-Henrik Groop	Folkhälsan Institute of Genetics, Folkhälsan Research Center, Helsinki, Finland.<br>Department of Nephrology, University of Helsinki and Helsinki University Hospital, Helsinki, Finland.<br>Research Program for Clinical and Molecular Metabolism, Faculty of Medicine, University of Helsinki, 00290, Helsinki, Finland.<br>Department of Diabetes, Central Clinical School, Monash University, Melbourne, Victoria, Australia.
**Diabetes Complications Research Centre, University College Dublin, Dublin Ireland**	
Ciarán Kennedy	Diabetes Complications Research Centre, Conway Institute, School of Medicine, University College Dublin, Dublin Ireland.

Ross Doyle	Diabetes Complications Research Centre, Conway Institute, School of Medicine, University College Dublin, Dublin Ireland.
Eoin Brennan	Diabetes Complications Research Centre, Conway Institute, School of Medicine, University College Dublin, Dublin Ireland.
Elena Giardini	Diabetes Complications Research Centre, Conway Institute, School of Medicine, University College Dublin, Dublin Ireland.
Darrell Andrews	Diabetes Complications Research Centre, Conway Institute, School of Medicine, University College Dublin, Dublin Ireland.
Denise Sadlier	Mater Misericordiae Hospital, Dublin, Ireland D07 K201.
Finian Martin	Diabetes Complications Research Centre, Conway Institute, School of Medicine, University College Dublin, Dublin Ireland.
Catherine Godson	Diabetes Complications Research Centre, Conway Institute, School of Medicine, University College Dublin, Dublin Ireland.
**University of Michigan School of Medicine, Ann Arbor, MI, USA**	
Viji Nair	Department of Medicine-Nephrology, University of Michigan School of Medicine, Ann Arbor, MI 48109, USA.
Damian Fermin	Department of Pediatrics-Nephrology, University of Michigan School of Medicine, Ann Arbor, MI 48109, USA.
Lalita Subramanian	Department of Medicine-Nephrology, University of Michigan School of Medicine, Ann Arbor, MI 48109, USA.
Matthias Kretzler	Department of Internal Medicine, University of Michigan, Ann Arbor, Michigan, USA.
**University of Pennsylvania, Perelman School of Medicine, Philadelphia, PA, USA.**	
Hongbo Liu	Renal, Electrolyte, and Hypertension Division, Department of Medicine, University of Pennsylvania, Perelman School of Medicine, Philadelphia, PA, USA.<br>Institute for Diabetes, Obesity, and Metabolism, University of Pennsylvania, Perelman School of Medicine, Philadelphia, PA, USA.<br>Department of Genetics, University of Pennsylvania, Perelman School of Medicine, Philadelphia, PA, USA
Katalin Susztak	Renal, Electrolyte, and Hypertension Division, Department of Medicine, University of Pennsylvania, Perelman School of Medicine, Philadelphia, PA, USA.<br>Institute for Diabetes, Obesity, and Metabolism, University of Pennsylvania, Perelman School of Medicine, Philadelphia, PA, USA.<br>Department of Genetics, University of Pennsylvania, Perelman School of Medicine, Philadelphia, PA, USA
**University of California San Diego, La Jolla, CA, USA**	
Rany M Salem	Herbert Wertheim School of Public Health and Human Longevity Science, University of California San Diego, La Jolla, CA, USA

Supplemental Methods

Participant recruitment and Biobank

The North Dublin Renal Biobank (NDRBB) was established as a biobank of samples from individuals with kidney disease with the aim of studying the molecular determinants of chronic kidney disease. Individuals who were undergoing evaluation because of kidney dysfunction were recruited and, following written, informed consent, biological samples and clinical data were collected. Available clinical data include demographic variables, details of kidney function, comorbidities, medication use as well as renal biopsy histology data. Data in relation to kidney function and kidney disease outcomes, generated as part of clinical follow-up were also prospectively collected.

Surplus kidney biopsy material, not required for histological diagnosis, was stored in *RNA-later* (Thermo Fisher) and stored at -80°C.

For this study only individuals with kidney disease in native kidneys, i.e. excluding those with prior kidney transplant, were included. Two independent cohorts of were chosen from our Biobank and where results were generated in one cohort, they were compared internally where possible using the alternative cohort to ensure consistency.

Human Renal Biopsies

Kidney biopsies and baseline clinical data were provided by the NDRBB. Clinical data generated were prospectively collected as part of routine follow-up over 5 years. Kidney function was assessed by estimated Glomerular Filtration Rate (eGFR), calculated using the Chronic Kidney Disease (CKD) Epidemiology Collaboration equation [1]. Percent tubulointerstitial fibrosis (%TIF) was assessed following histological assessment of trichrome-stained kidney biopsy by a kidney pathologist. Progressive CKD was defined as either doubling of serum creatinine, a validated surrogate endpoint [2], or the development of end-stage kidney disease. Stable kidney disease was defined based on follow-up creatinine measurements being within ± 10% of the participant's measurement at study entry. Participants in the study provided informed written consent prior to participation. Ethical approval for this work was granted by the Beaumont Hospital Ethics (Medical Research) Committee. All data were processed in line with the General Data Protection Regulation of the European Union [3].

RNA extraction, quantification, and quality assessment

At the time of processing, the frozen tissue was allowed to reach room temperature before homogenization, performed using a Polytron tissue homogenizer. Homogenized samples were then processed using a column extraction kit (Qiagen RNeasy Mini Kit) according to the manufacturer's instructions to produce purified RNA. The RNA eluted was quantified using a spectrophotometer (Nanodrop 2000 ®, Thermo Scientific). The

quality of the eluted RNA was examined using the RNA Nano 6000 LabChip kit (Agilent Technologies). This assessment identifies the RNA Integrity Number (RIN), samples with an RIN ≥ 8 were selected for sequencing.

RNA sequencing

Samples were sequenced at either 100 base-pair paired-end reads using the BGISEQ-500 platform (Cohort 1, eGFR, %TIF correlation) or 50 base-pair single-end reads using the Illumina HiSeq 2000 platform (Cohort 2, Progressive disease analysis) at the Beijing Genomics Institute. Raw transcript reads were aligned to the human genome, hg19 using the STAR aligner within Partek Flow® software according to the manufacturer's specification. Following alignment, genes with zero read counts in more than one sample were removed and the remaining read counts were quantile normalized. All further analyses were performed using R.

Differentially expressed transcripts in progressive versus stable CKD individuals were identified using the limma package in R, following adjustment for age and sex. Enrichr-KG and Ingenuity Pathway Analysis (Qiagen) was used to examine the top pathways represented within our dataset, and for data visualization. A false discovery rate (FDR) adjusted p-value of <0.05 was deemed statistically significant. Venn diagrams of gene / pathway overlap were generated using jvenn [4].

Correlation Analysis

We examined the correlation of normalized gene expression values from our first cohort with clinically relevant attributes. A linear regression model was used to examine the correlation between gene expression and clinico-pathological variables of eGFR and %TIF, with age and sex used as co-variables, using the lm/cor.test functions in R. A false discovery rate (FDR) adjusted p-value cut-off of < 0.05 was used to identify statistically significant findings.

Differential Expression Analysis

Using a second, independent cohort of patients (cohort 2), differentially expressed genes between individuals who had developed progressive kidney disease over their period of follow-up and those who had experienced stable kidney function were measured.

Differentially expressed genes were identified using the limma package [5] in R. Our analyses included adjustment for patient age and sex and an FDR adjusted p-value cut-off of <0.05 was used to identify statistically significant findings. We generated lists of genes which were upregulated in the setting of progressive kidney disease and genes which were downregulated in this context.

Pathway Analysis

We used the Enrichr KG web service (Go Biological Processes and Reactome databases) and Ingenuity Pathway Analysis (Qiagen) to examine the top pathways

represented within our dataset, and for data visualization. An FDR-adjusted p-value cut-off of $p < 0.05$ was deemed statistically significant.

Analysis of publicly available Single-cell datasets.

Pre-processed, aggregated and clustered single-nucleus RNA-seq data were downloaded from the KPMP kidney tissue atlas repository (KPMP Atlas Explorer v1.3 data, DOI 10.48698/yyvc-ak78) [6]. Healthy control and CKD samples were used for further analysis and visualization. Visualization was performed in R using the Seurat [7] and ggplot2 [8] packages. Differential expression analysis between healthy reference and CKD samples was performed using Wilcoxon rank-sum testing with Bonferroni correction within Seurat.

Pre-processed and plotted data from single-nucleus RNA-sequencing from early human diabetic nephropathy and healthy control samples [9] were downloaded using the web-based Kidney Interactive Transcriptomics tool (http://humphreyslab.com/SingleCell/, accessed 18th December 2023, figure published with permission)

FRMD3 in vitro studies

HK-2 cell culture

Human proximal tubule epithelial (HK-2) cells (ATCC) were maintained in a 1:1 solution of DMEM (without glucose), (Gibco) and F12 Nutrient Mix (Gibco) supplemented with 100 Units/mL penicillin, 100 µg/mL streptomycin (Penicillin-Streptomycin, Gibco), 4.5 mM L-glutamine (Gibco), 10 ng/mL recombinant human epidermal growth factor (Sigma Aldrich), 36 ng/mL hydrocortisone (Sigma Aldrich), 10µg/mL insulin, 5.5 µg/mL transferrin and 5 ng/mL selenium (ITS, Sigma Aldrich). Cells were maintained in a humidified incubator at 37°C, 5% CO_2. Cells were passaged at 70-80% confluence and media were changed every 48-72 hours.

Recombinant transforming growth factor-β1 (TGF-β1, PromoCell) was used to induce a fibrotic response in renal cells (2.5ng/ml; 48h), with diluent (0.1% (w/v) Bovine serum albumin in DPBS) used as a vehicle control.

Lentiviral Particle Production

Lentivirus particles were produced by transfecting Human Embryonic Kidney cells (HEK293T, ATCC) cells in a T75 flask at 70% confluency with the target vector, a vector expressing a lentiviral envelope protein (pCMV-VSV-G) and a lentiviral packaging plasmid (psPAX2). pCMV-VSV-G was a gift from Bob Weinberg (MIT, Addgene, plasmid # 8454) [10]. psPAX2 was a gift from Didier Trono (EPFL, Addgene plasmid # 12260)

For shRNA knockdown, a commercially available shRNA targeting FRMD3 was used (TRCN0000161190, Sigma Aldrich, Mission shRNA). A scrambled control shRNA

vector (MISSION pLKO.1-puro non-mammalian shRNA Control, Sigma Aldrich, SHC002) was used as a negative control for knockdown studies.

FRMD3-V5 cDNA was sub-cloned into a commercial lentiviral expression vector (CD510B-1, System Biosciences) upstream of the V5 tag using EcorI restriction site. Empty backbone vector (CD510B-1) was used as a negative control for mass spectrometry analysis in tagged overexpressing cells.

Stable cell lines were selected in the presence of 2.5μg/mL puromycin for 7 days before use or cryopreservation.

qRT-PCR

Total RNA was isolated from HK-2 cells using an E.Z.N.A. Total RNA Kit 1 (Omega Bio-tek). Real-Time Quantitative Reverse Transcription PCR was performed to determine *FRMD3* expression level before and after knock-down. TaqMan probes for *FRMD3* were supplied as a pre-optimized single tube primer/probe Gene Expression Assay (Applied Biosystems) and the assay run using a QuantStudio 7 Flex Real-Time PCR system (Applied Biosystems). TaqMan probes for β-actin were used as an endogenous control for normalization of the target genes. Results were analyzed using the comparative Ct method of analysis. Results were plotted in GraphPad Prism and significance calculated by Wilcoxon Rank Sum Test. N = 3 biological replicates.

Immunofluorescence imaging of FRMD3-V5 expressing HK-2 cells.

FRMD3-V5 overexpressing HK-2 cells were seeded in 8-well chamber slides (Ibidi) and grown to the desired level of confluency. Cells were fixed in 4% (v/v) paraformaldehyde in PHEM buffer (60mM PIPES, 25mM HEPES, 10mM EGTA, and 4mM MgSO4·7H20) for 10 min at room temperature and blocked for 1 h at room temperature, in blocking solution containing 5% (v/v) goat serum in PBS. Cells were incubated in primary antibody at 4 °C overnight. Following incubation, cells were washed with PHEM buffer and incubated overnight at 4 °C with their corresponding fluorescence-conjugated secondary antibody (Alexa Fluor 488/568 conjugated anti mouse / rabbit, Thermo Fisher, 1:200-500). Slides were again washed with PHEM buffer and incubated with Rhodamine-conjugated phalloidin (Thermo Fisher, 1:400) and Hoeschst33342 (Thermo Scientific, 1:1000) as required and subsequently washed with PHEM buffer before mounting. 2D images were acquired using a Zeiss 200M inverted fluorescence microscope, while confocal z-stacks were captured using a Zeiss LSM800 confocal microscope in airyscan mode. Brightness and contrast adjustments, z-projection, background subtraction and linear spectral unmixing were performed using Zeiss Zen or ImageJ as required.

Western Blot

Western blot analysis of HK-2 cell lysates was performed to determine protein abundance. Normalized protein extract was resolved by SDS-PAGE. Proteins were then

transferred onto Immobilon P-transfer membranes (Millipore), blocked with PBS-T (137mM NaCl, 2.7mM KCL, 10mM Na_2HPO_4, 1.8mM KH_2PO_4, and 0.1% (v/v) Tween 20) supplemented with 5% (w/v) non-fat dried milk and then incubated with the appropriate primary antibody as listed below. Membranes were subsequently incubated with horseradish peroxidase-linked secondary antibodies (New England Biolabs). Blots were developed using enhanced chemiluminescence reagents (Advansta) and imaged using a Vilber Fusion FX 7. Densitometry analysis was performed using ImageJ and plotted using GraphPad Prism. Significance was determined using Sidak's multiple comparison testing following two-way ANOVA. N = 3 biological replicates.

Proteomic Analysis

To investigate the *FRMD3* interactome, HK-2 cell lines overexpressing *FRMD3*-V5, or the empty CD510B-1 vector, were grown to confluence and lysed in a hypertonic lysis buffer containing detergent (1%(v/v) Triton X-100), protease and phosphatase inhibitor cocktails. Protein concentration was assayed by Bradford assay and 700µg of protein was incubated with 20µL of anti-V5 antibody conjugated agarose beads for 90 mins at 4ºC on a rotary shaker. On-bead protein digestion and mass spectrometry (MS) analysis was performed using a Q-Exactive mass spectrometer/Ultimate Ultra3000 chromatography system (Thermo Scientific, Germany). MaxQuant was used to analyze raw data from the LC-MS/MS for protein quantification. Three biological replicates with two technical replicates each were analyzed. Label-free quantification (LFQ) values were used for protein quantification. Student's t-test was used to test for variance between samples and cutoffs were applied to get a list of specific interactors: p-value < 0.05, *FRMD3*-V5 antiV5: EV anti-V5 ratio ≥ 2.

Mitochondrial Function/Caspase Assays

The effects of *FRMD3* knockdown and overexpression on mitochondrial function and metabolic activity in renal epithelial cells was investigated in HK-2 cells transduced with a lentivirus encoding either a shRNA targeting *FRMD3* mRNA or control non-targeting shRNA. Absolute mitochondrial content was measured using Mitotracker Green (Invitrogen). Fluorescence was measured on a spectrophotometer (Spectra M2, Molecular Devices, excitation 485nm, emission 530nm). MTT (3-(4,5-dimethylthiazol-2-yl)-2,5-diphenyltetrazolium bromide) was used to measure metabolic activity. MTT (1.25mg/ml) was added to cells (100µL/well) and incubated at 37ºC for two hours. Media was aspirated and cells were washed with 150µL PBS. The PBS was then aspirated, and the formazan product dissolved with 100µL of DMSO per well for 10 mins on a rotary shaker. The absorbance of each well was then measured at 570nm (Spectra M2, Molecular Devices) and quantitated relative to absolute mitochondrial content (calculated by Mitotracker Green assay). A resazurin assay was used to measure dehydrogenase enzyme (NADH/NADPH) activity. Resazurin was added to HK-2 cells (100µL/well) and incubated at 37ºC for two hours. Fluorescence was recorded using the Spectra M2 (Molecular Devices, excitation 560nm, emission 590nm) and made relative to absolute mitochondrial content (calculated by Mitotracker Green assay).

Caspase 3/7 activity in HK-2 cells was measured using Ac-DEVD-AFC compound.

Ac-DEVD-AFC stock solutions were prepared as per the manufacturer's protocol (Enzo Life Sciences). Cells were lysed in ice-cold caspase assay lysis buffer (CALB, 100µL: Glycerol 10% (v/v), CHAPS 0.5% (v/v), 0.5 mM EDTA (pH8), 0.1 mM PMSF and 5mM DTT). For analysis, 80µL of cell lysate was added per well of a black 96-well plate. To 1mL of caspase assay lysis buffer (CALB), 1µL of Ac-DEVD-AFC (100mM) was added to give a final concentration of 100µM. Fluorescence was read on a spectrophotometer (Spectra M2, Molecular Devices) in a kinetic manner for 2h (1 min intervals, 37ºC, auto-mixing 1 sec before reading, excitation 400nm, emission 505nm).

Primary antibodies used in this study:

Immunofluorescence

Antibody Target	Source	Cat Number	Dilution
**V5 (Mouse)**	Invitrogen	R96025	1:500
**V5 (Rabbit)**	Invitrogen	MA532053	1:500
**P120 Catenin**	BD Transduction Laboratories	610133	1:250
**TJP1**	Invitrogen	61-7300	1:250

Western Blot

Antibody Target	Source	Cat Number	Dilution
**E-Cadherin**	BD Transduction Laboratories	610181	1:1,000
**Alpha-Tubulin**	Abcam	ab7291	1:20,000

References

1. Levey, A.S., et al., *A new equation to estimate glomerular filtration rate.* Ann Intern Med, 2009. **150**(9): p. 604-12.
2. Jun, M., et al., *Assessing the Validity of Surrogate Outcomes for ESRD: A Meta-Analysis.* J Am Soc Nephrol, 2015. **26**(9): p. 2289-302.
3. *Regulation (EU) 2016/679 of the European Parliament and of the Council of the 27 April 2016 on the protection of natural persons with regard to the processing of personal data and on the free movement of such data, and repealing Directive 95/46/EC (General Data Protection Regulation).* Official Journal of the Eruopean Union. **L199**: p. 1-88.
4. Bardou, P., et al., *jvenn: an interactive Venn diagram viewer.* BMC Bioinformatics, 2014. **15**(1): p. 293.
5. Ritchie, M.E., et al., *limma powers differential expression analyses for RNA-sequencing and microarray studies.* Nucleic Acids Res, 2015. **43**(7): p. e47.
6. *Kidney Precision Medicine Project, Aggregated, clustered single-nucleus RNA-seq data used in the KPMP Atlas Explorer v1.3. Kidney Precision Medicine Project.* https://doi.org/10.48698/yyvc-ak78. 2021.
7. Stuart, T., et al., *Comprehensive Integration of Single-Cell Data.* Cell, 2019. **177**(7): p. 1888-1902.e21.

8. Wickham, H., *Ggplot2: Elegant graphics for data analysis*. 2 ed. 2016, Basel, Switzerland: Springer International Publishing.
9. Wilson, P.C., et al., *The single-cell transcriptomic landscape of early human diabetic nephropathy.* Proc Natl Acad Sci U S A, 2019. **116**(39): p. 19619-19625.
10. Stewart, S.A., et al., *Lentivirus-delivered stable gene silencing by RNAi in primary cells.* Rna, 2003. **9**(4): p. 493-501.

Supplemental Figure Legends:

S1: Correlation of Clinical variables of kidney disease within the NDRBB cohorts.

Correlation analysis of clinical variables of renal damage and function between (A) Glomerulosclerosis with % Tubulointerstitial Fibrosis (Pearson r = 0.75, p = 1.001 x 10^{-9}), (B) eGFR with % Tubulointerstitial Fibrosis (Pearson r=-0.61, p = 5.274 x 10^{-6}) and (C) eGFR with Glomerulosclerosis (Pearson r=-0.45, p = 0.001583) shows strong correlations between each in our discovery cohort (n = 24).

S2: Enriched terms in genes correlated both positively and negatively with either eGFR or %TIF in batient biopsy samples.

Bar charts of top 10 Enrichment results from Enrichr KG using the Go Biological Processes (GO: codes) and Reactome (R-HAS- Codes) databases. Gene sets used were genes correlating with estimated glomerular Filtration Rate (eGFR) and % Tubulointerstitial Fibrosis on biopsy (%TIF). Terms were filtered for Q-score < 0.05 and sorted by combined score.

S3: Gene set enrichment analysis highlights the overlapping processes between disease severity and future disease progression.

Gene set enrichment analysis was performed on gene sets correlating with disease severity in patient renal biopsies (n = 24) using the Enrichr KG platform and the Reactome and Go Biological Processes databases, taking the top 10 resulting processes by Q-score. Genes associated with more severe disease (A) (positive correlation with %TIF and negative correlation with eGFR, 359 genes) showed enrichment for terms relating to the RUNX family of transcription factors, crucial mediators of immune cell identity and inflammatory signalling. Genes associated with less severe disease (B) (negative correlation with %TIF and positive correlation with eGFR, 574 genes) were enriched in terms related to mitochondrial function and energy production.
Similarly, genes differentially expressed in patients whose CKD would progress in severity at a follow-up visit 60-months post enrolment (progressive CKD) vs patients whose disease remained stable at this follow-up visit (stable CKD) were examined in a separate patient cohort (n = 17). Gene set enrichment analysis of these genes was then compared with the enriched terms for genes correlating with eGFR and with %

Tubulointerstitial fibrosis. Again, the Enrichr KG platform and the Reactome and Go Biological Processes databases were used, with the top 50 processes from each dataset by combined score used for comparison (100 total terms each).
Looking at genes upregulated in progressive CKD, we can see a much larger overlap between enriched processed in genes upregulated in progressive CKD and genes positively correlated with %TIF (55/100 processes in common), vs enriched processes in genes upregulated in progressive CKD and genes negatively correlated with eGFR (just 8/100 in common).
This trend was noticeably less distinct when interrogating gene set enrichment of genes downregulated in progressive CKD, sharing just 24/100 terms with terms enriched in genes negatively correlated with %TIF and sharing just 17 with genes positively correlating with eGFR.
Terms were filtered for Q-score < 0.05 and sorted by combined score.

S4: FRMD3 expression in microarray analysis of microdiscected kidney biopsy samples.

Microarray analysis of microdissected surgical nephrectomies (n = 432) shows lower levels of FRMD3 expression in the Tubule compartment (A) in CKD and DKD compared to healthy control samples. A strong correlation is also seen between FRMD3 expression and eGFR (cor = 0.47, p = 2 x 10^{-16}) and a strong negative correlation is also observed with fibrosis within this compartment (cor = -0.672, p = 2 x 10^{-16}). This however is not seen in the glomerular compartment (B), though similar but much weaker trends in correlation were observed. This suggests that FRMD3 correlates with kidney disease severity in a cell-type specific manner.
Con = Control, CKD = Chronic Kidney Disease, DKD = Diabetic Kidney Disease, DM = Diabetes Mellitus, HTN = Hypertension

S5: ***FRMD3 is enriched at the periphery of HK-2 cells at high density.***

(A) In confluent FRMD3-V5 expressing HK-2 proximal tubule cells, FRMD3 is enriched at the periphery of the cell, alongside F-actin, p120 catenin (a marker of adherens junctions) and Zo-1 (a marker of tight junctions) (100x magnification, scale bars 20µm).
(B) Laser-scanning confocal microscopy of V5-tagged FRMD3 overexpressing HK-2 cells shows Zo-1 (a marker of tight junctions, red) localized largely towards the apical surface at cell-cell contact points (yellow arrows) and partially co-localizing with FRMD3-V5 (Green). The main image (Scale bar 20µm) represents maximum projection of the sample, the yellow crosshairs represent the area taken as orthogonal slices displayed to the right (YZ) and bottom (XZ) of the main image. The red box around the XZ orthogonal sections mark the area enlarged below the main image set.

S6: ***Full length western blot images used for densitometry in Figure 5.***

(A.) Wild-type (WT) HK-2 proximal tubule cells as well HK-2 cells expressing an shRNA against *FRMD3* (KD) as well as HK-2 cells expressing a scrambled non-targeting control shRNA (SCR) were treated with 2.5ng/ml TGFβ-1 or vehicle diluent for 48 hours and assayed for levels of E-cadherin, with alpha-tubulin used as a loading control. Full length blots are displayed for each of 3 biological replicates. Where lanes

have not been labelled, they are not applicable to the data presented in this manuscript. (B.) Graphical representation of the protein ladder used in (A.) (Biorad, Precision Plus Dual Colour Protein Ladder)

Supplemental Figure S7: FRMD3 knockdown does not result in significant alterations in long-chain fatty acid oxidation in HK-2 kidney proximal tubule cells.

Wild-type (WT) HK-2 proximal tubule cells as well HK-2 cells expressing an shRNA against FRMD3 (KD) as well as HK-2 cells expressing a scrambled non-targeting control shRNA (SCR) were subject to the Agilent Seahorse Palmitate Oxidation Stress Test – Advanced Assay. In conditions of limiting glucose, pyruvate and glutamine, and in the presence of saturating L-carnitine, cells were treated with palmitate-BSA conjugate (6:1) or fatty-acid-free BSA control subject to sequential injections of Etomoxir (or media control), oligomycin, FCCP and rotenone/Antimycin A. Oxygen consumption rate (OCR) and Extracellular Acidification Rate (ECAR) were monitored throughout with the Seahorse XF Pro instrument, and results calculated using the Agilent Seahorse Data Analytics Platform. No significant differences were observed between WT, shSCR and shFRMD3 cells with respect to maximal respiration (the maximum oxygen consumption rate achieved following uncoupling by FCCP) (A) or Acute response (Change in OCR with respect to baseline upon addition of Etomoxir) (B).

Supplemental Figures:

S1.

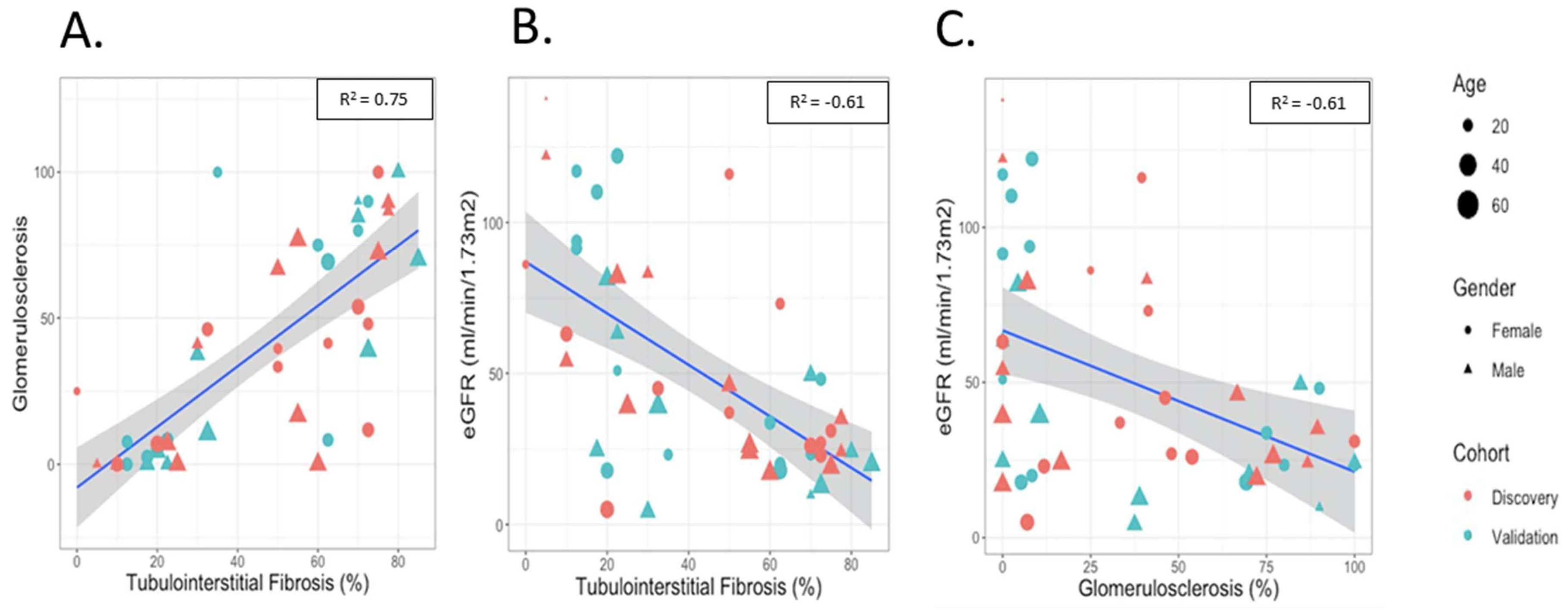

A.
R² = 0.75
Glomerulosclerosis
Tubulointerstitial Fibrosis (%)
B.
R² = -0.61
eGFR (ml/min/1.73m2)
Tubulointerstitial Fibrosis (%)
C.
R² = -0.61
eGFR (ml/min/1.73m2)
Glomerulosclerosis (%)
Age
20
40
60
Gender
Female
Male
Cohort
Discovery
Validation

S2.

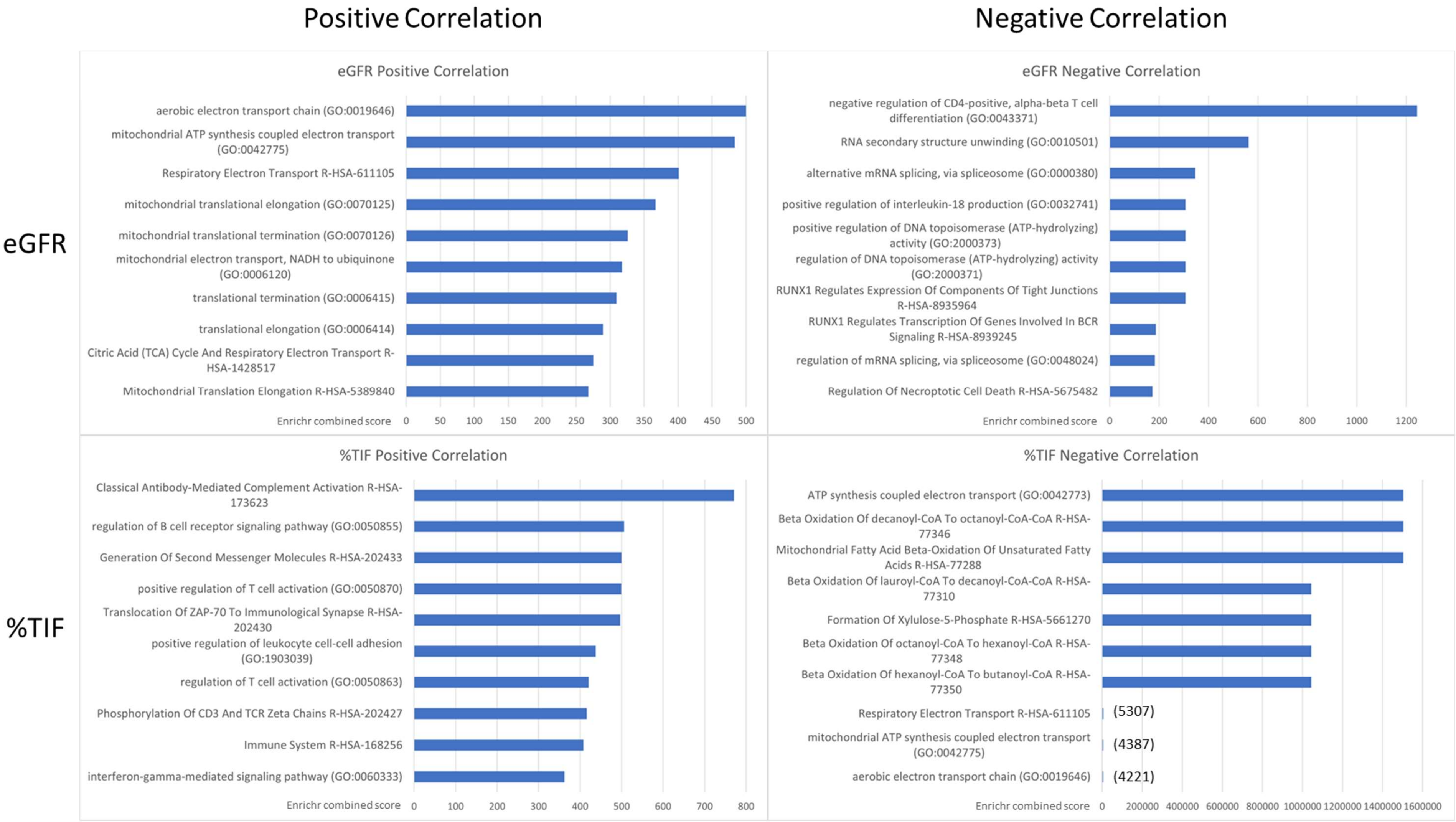
Positive Correlation
Negative Correlation
eGFR
%TIF
eGFR Positive Correlation
aerobic electron transport chain (GO:0019646)
mitochondrial ATP synthesis coupled electron transport (GO:0042775)
Respiratory Electron Transport R-HSA-611105
mitochondrial translational elongation (GO:0070125)
mitochondrial translational termination (GO:0070126)
mitochondrial electron transport, NADH to ubiquinone (GO:0006120)
translational termination (GO:0006415)
translational elongation (GO:0006414)
Citric Acid (TCA) Cycle And Respiratory Electron Transport R-HSA-1428517
Mitochondrial Translation Elongation R-HSA-5389840
Enrichr combined score
0 50 100 150 200 250 300 350 400 450 500
eGFR Negative Correlation
negative regulation of CD4-positive, alpha-beta T cell differentiation (GO:0043371)
RNA secondary structure unwinding (GO:0010501)
alternative mRNA splicing, via spliceosome (GO:0000380)
positive regulation of interleukin-18 production (GO:0032741)
positive regulation of DNA topoisomerase (ATP-hydrolyzing) activity (GO:2000373)
regulation of DNA topoisomerase (ATP-hydrolyzing) activity (GO:2000371)
RUNX1 Regulates Expression Of Components Of Tight Junctions R-HSA-8935964
RUNX1 Regulates Transcription Of Genes Involved In BCR Signaling R-HSA-8939245
regulation of mRNA splicing, via spliceosome (GO:0048024)
Regulation Of Necroptotic Cell Death R-HSA-5675482
Enrichr combined score
0 200 400 600 800 1000 1200
%TIF Positive Correlation
Classical Antibody-Mediated Complement Activation R-HSA-173623
regulation of B cell receptor signaling pathway (GO:0050855)
Generation Of Second Messenger Molecules R-HSA-202433
positive regulation of T cell activation (GO:0050870)
Translocation Of ZAP-70 To Immunological Synapse R-HSA-202430
positive regulation of leukocyte cell-cell adhesion (GO:1903039)
regulation of T cell activation (GO:0050863)
Phosphorylation Of CD3 And TCR Zeta Chains R-HSA-202427
Immune System R-HSA-168256
interferon-gamma-mediated signaling pathway (GO:0060333)
Enrichr combined score
0 100 200 300 400 500 600 700 800
%TIF Negative Correlation
ATP synthesis coupled electron transport (GO:0042773)
Beta Oxidation Of decanoyl-CoA To octanoyl-CoA-CoA R-HSA-77346
Mitochondrial Fatty Acid Beta-Oxidation Of Unsaturated Fatty Acids R-HSA-77288
Beta Oxidation Of lauroyl-CoA To decanoyl-CoA-CoA R-HSA-77310
Formation Of Xylulose-5-Phosphate R-HSA-5661270
Beta Oxidation Of octanoyl-CoA To hexanoyl-CoA R-HSA-77348
Beta Oxidation Of hexanoyl-CoA To butanoyl-CoA R-HSA-77350
Respiratory Electron Transport R-HSA-611105
(5307)
mitochondrial ATP synthesis coupled electron transport (GO:0042775)
(4387)
aerobic electron transport chain (GO:0019646)
(4221)
Enrichr combined score
0 200000 400000 600000 800000 1000000 1200000 1400000 1600000

S3.

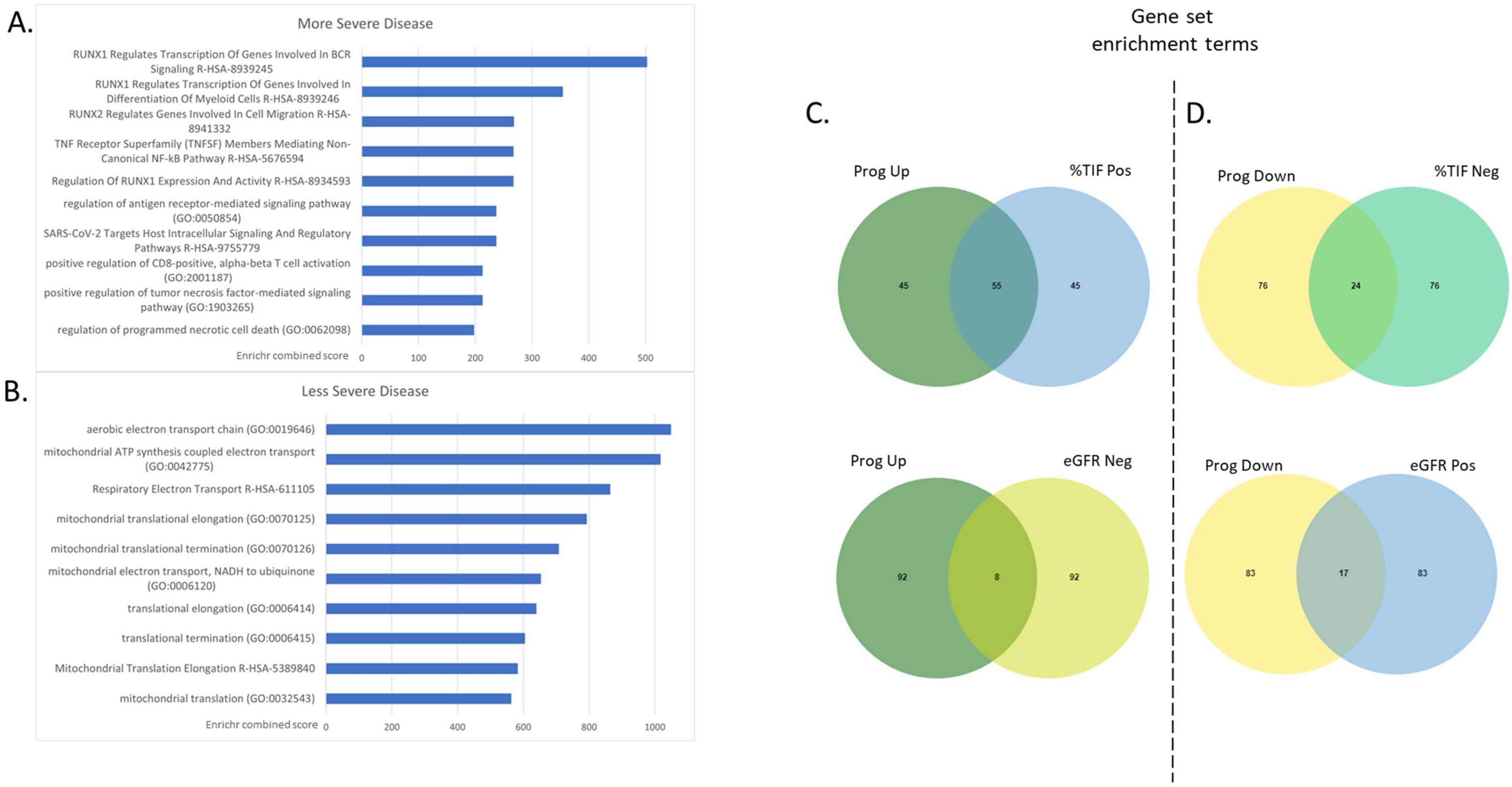
A.
More Severe Disease
RUNX1 Regulates Transcription Of Genes Involved In BCR Signaling R-HSA-8939245
RUNX1 Regulates Transcription Of Genes Involved In Differentiation Of Myeloid Cells R-HSA-8939246
RUNX2 Regulates Genes Involved In Cell Migration R-HSA-8941332
TNF Receptor Superfamily (TNFSF) Members Mediating Non-Canonical NF-kB Pathway R-HSA-5676594
Regulation Of RUNX1 Expression And Activity R-HSA-8934593
regulation of antigen receptor-mediated signaling pathway (GO:0050854)
SARS-CoV-2 Targets Host Intracellular Signaling And Regulatory Pathways R-HSA-9755779
positive regulation of CD8-positive, alpha-beta T cell activation (GO:2001187)
positive regulation of tumor necrosis factor-mediated signaling pathway (GO:1903265)
regulation of programmed necrotic cell death (GO:0062098)
Enrichr combined score
0
100
200
300
400
500
B.
Less Severe Disease
aerobic electron transport chain (GO:0019646)
mitochondrial ATP synthesis coupled electron transport (GO:0042775)
Respiratory Electron Transport R-HSA-611105
mitochondrial translational elongation (GO:0070125)
mitochondrial translational termination (GO:0070126)
mitochondrial electron transport, NADH to ubiquinone (GO:0006120)
translational elongation (GO:0006414)
translational termination (GO:0006415)
Mitochondrial Translation Elongation R-HSA-5389840
mitochondrial translation (GO:0032543)
Enrichr combined score
0
200
400
600
800
1000
Gene set enrichment terms
C.
Prog Up
%TIF Pos
45
55
45
Prog Up
eGFR Neg
92
8
92
D.
Prog Down
%TIF Neg
76
24
76
Prog Down
eGFR Pos
83
17
83

S4.

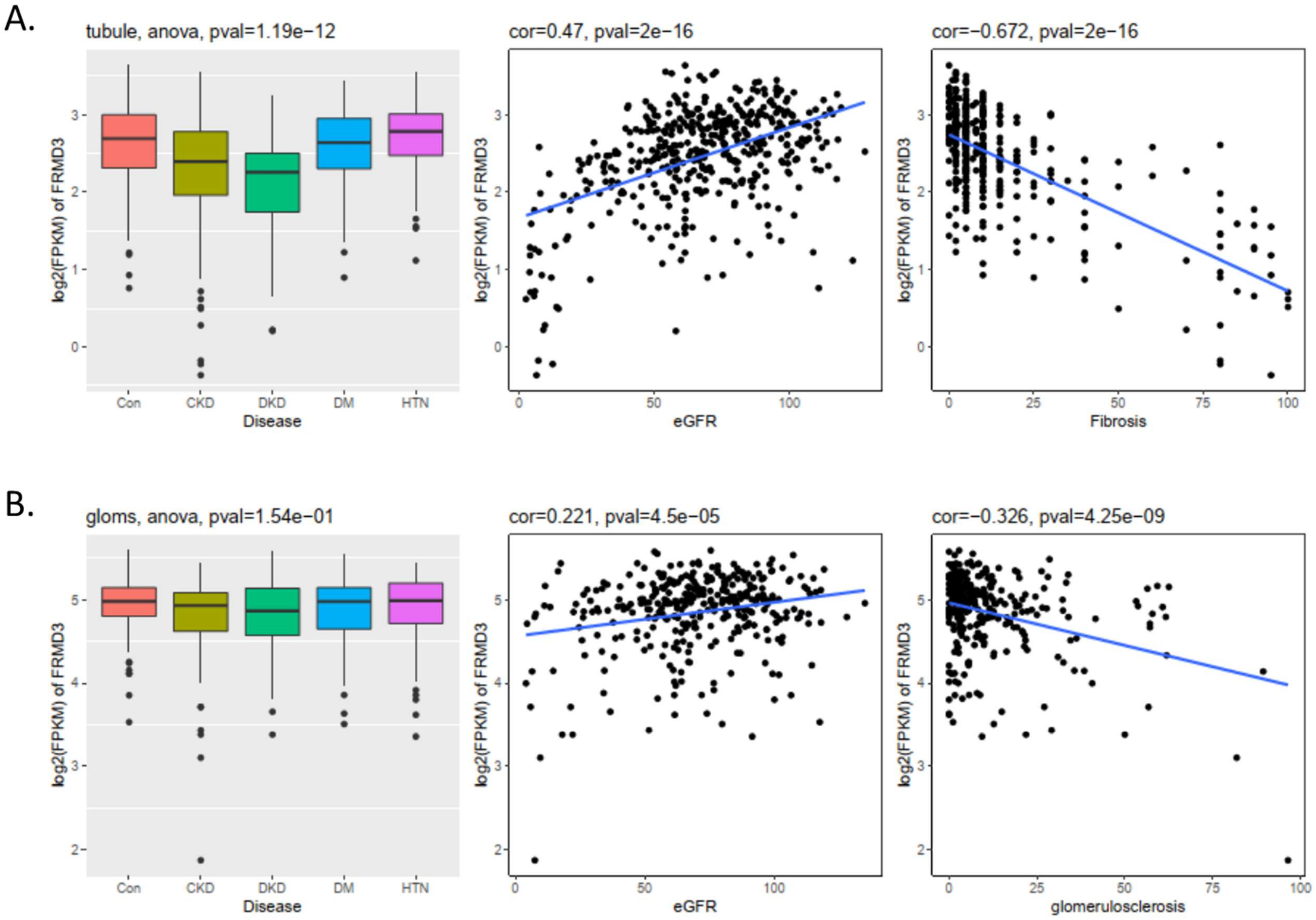
A.
tubule, anova, pval=1.19e−12
log2(FPKM) of FRMD3
Con
CKD
DKD
DM
HTN
Disease
cor=0.47, pval=2e−16
eGFR
cor=−0.672, pval=2e−16
Fibrosis
B.
gloms, anova, pval=1.54e−01
Disease
cor=0.221, pval=4.5e−05
eGFR
cor=−0.326, pval=4.25e−09
glomerulosclerosis

S5.

A.

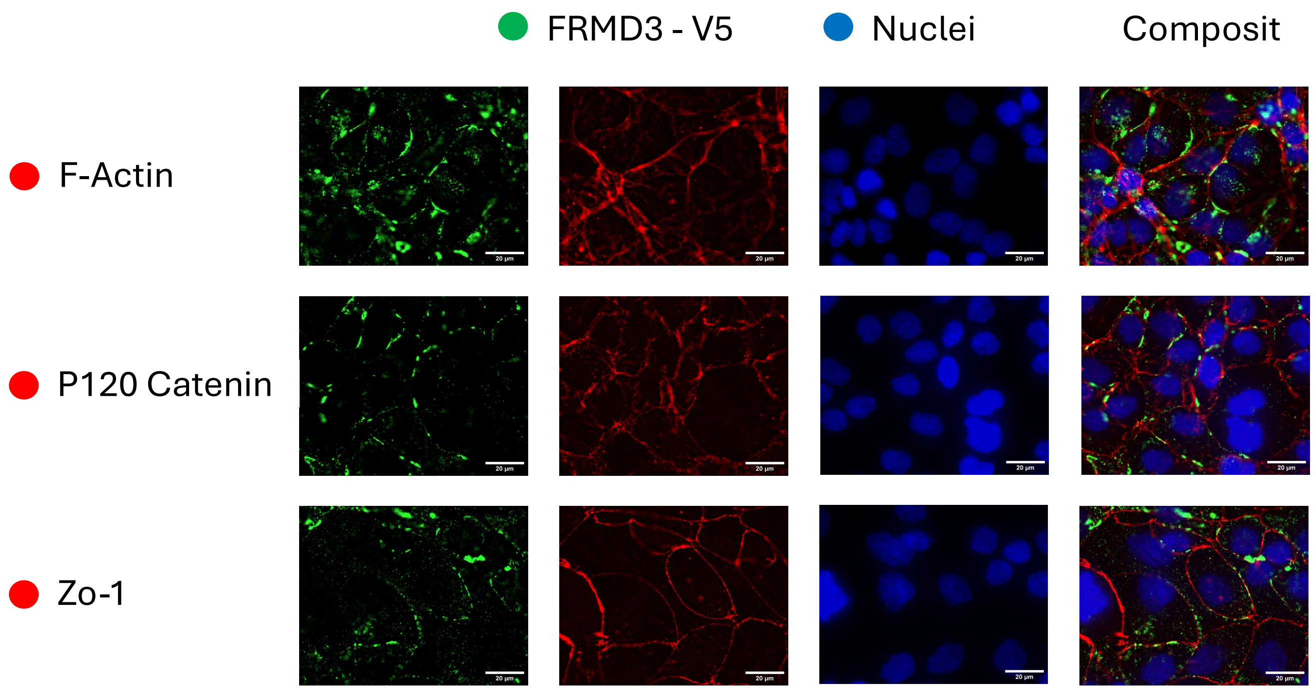

FRMD3 - V5
Nuclei
Composit
F-Actin
P120 Catenin
Zo-1

B.

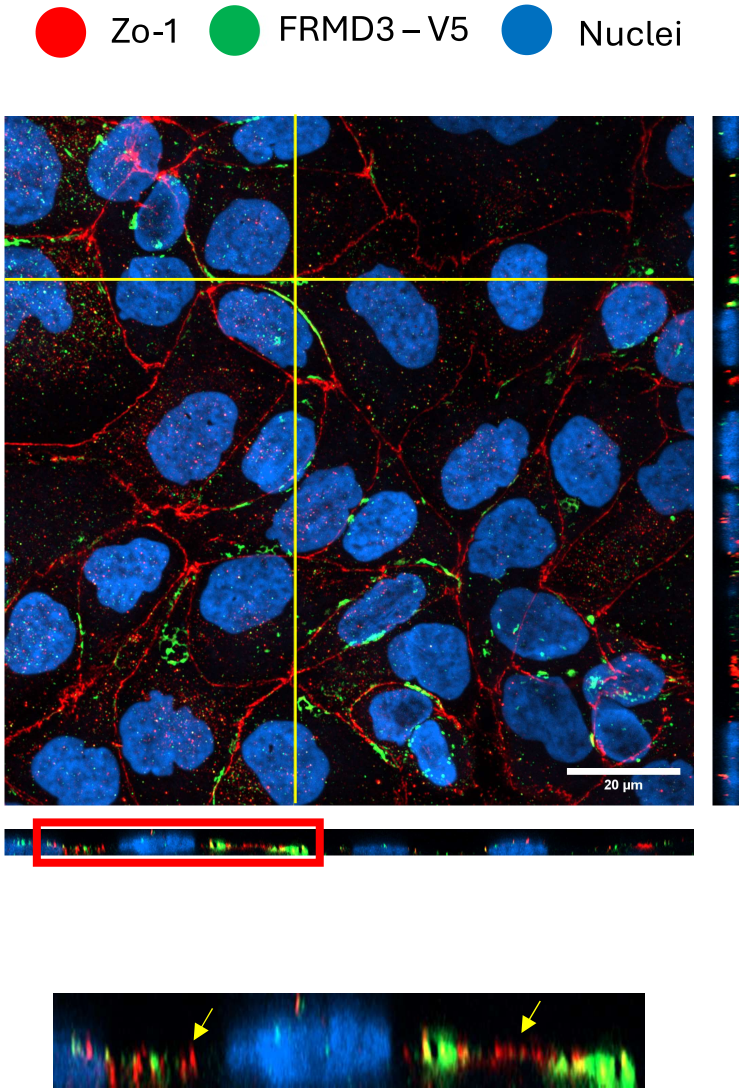

Zo-1
FRMD3 – V5
Nuclei
20 µm

S6.

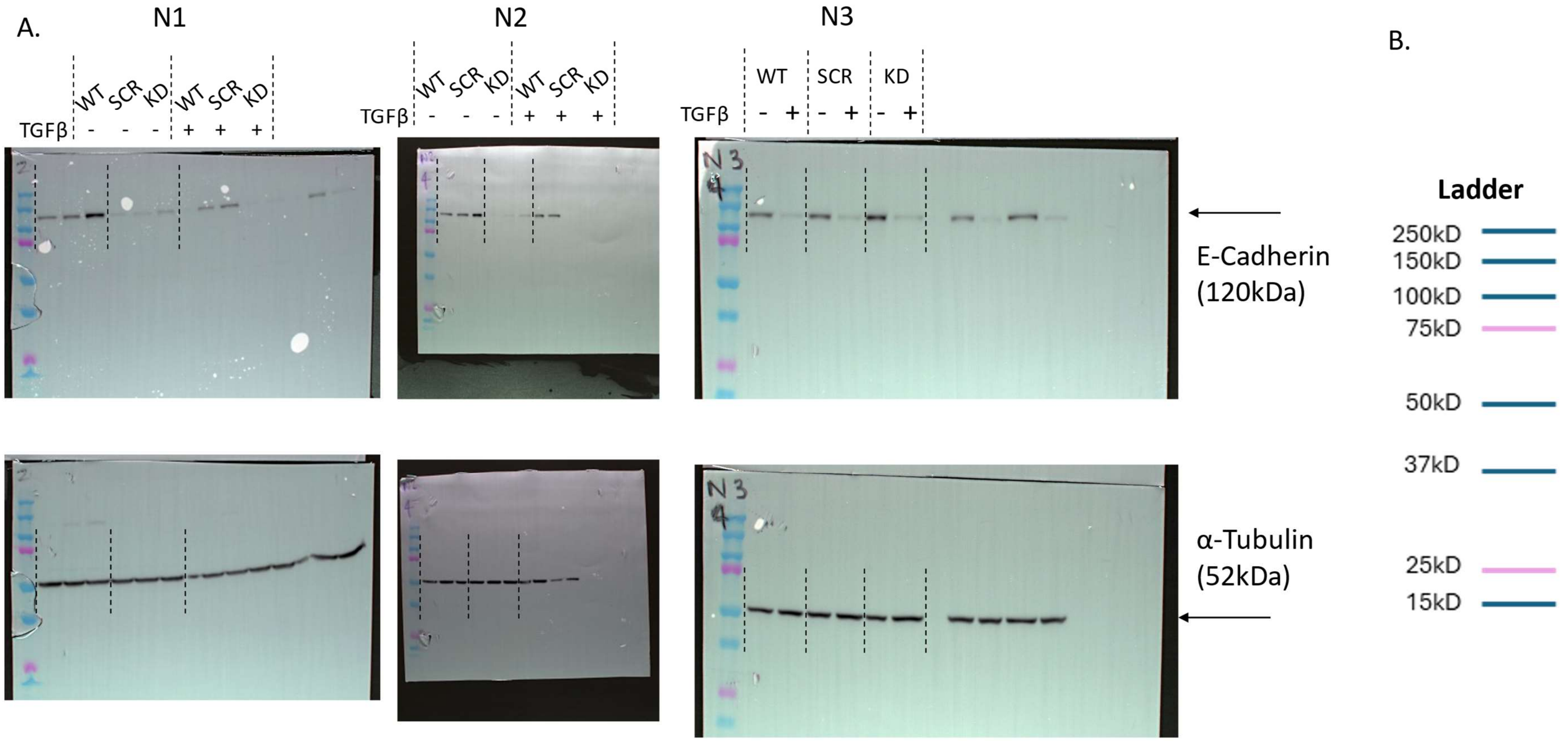
A.
N1
WT SCR KD WT SCR KD
TGFβ - - - + + +
N2
WT SCR KD WT SCR KD
TGFβ - - - + + +
N3
WT SCR KD
TGFβ - + - + - +
N3
E-Cadherin
(120kDa)
α-Tubulin
(52kDa)
B.
Ladder
250kD
150kD
100kD
75kD
50kD
37kD
25kD
15kD

S7.

A.

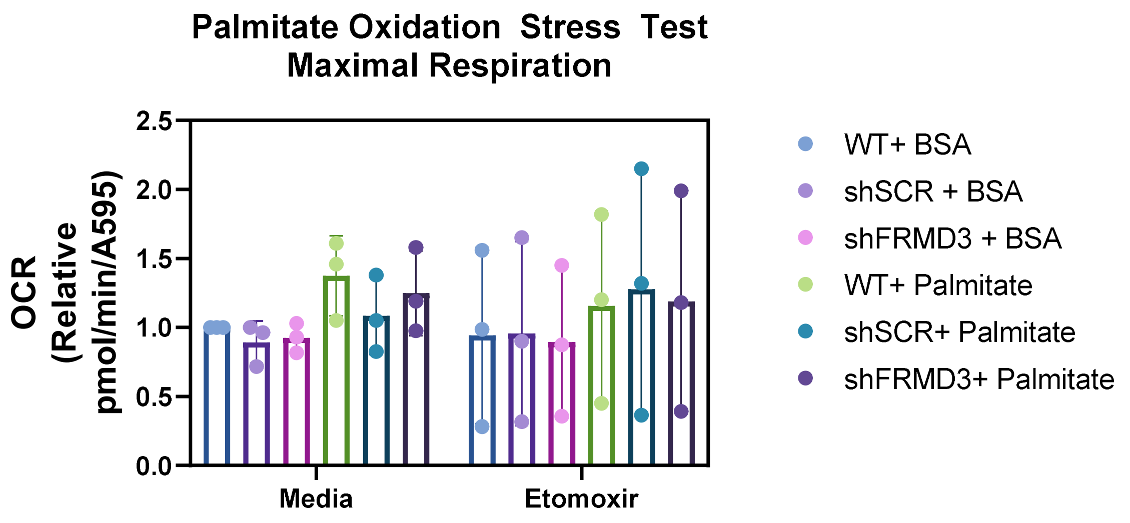

Palmitate Oxidation Stress Test
Maximal Respiration
OCR
(Relative
pmol/min/A595)
2.5
2.0
1.5
1.0
0.5
0.0
Media
Etomoxir
WT+ BSA
shSCR + BSA
shFRMD3 + BSA
WT+ Palmitate
shSCR+ Palmitate
shFRMD3+ Palmitate

B.

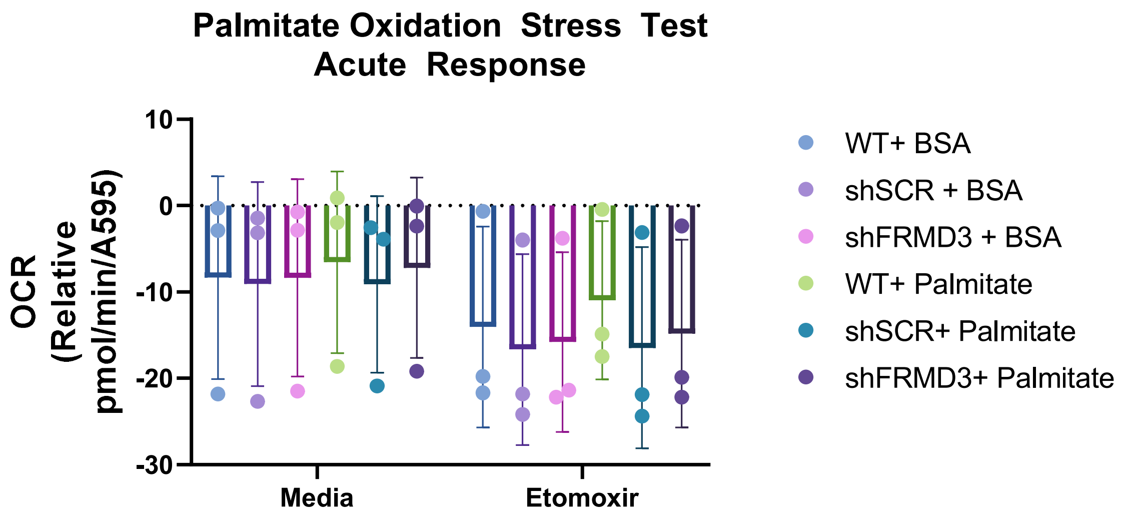

Palmitate Oxidation Stress Test
Acute Response
OCR
(Relative
pmol/min/A595)
10
0
-10
-20
-30
Media
Etomoxir
WT+ BSA
shSCR + BSA
shFRMD3 + BSA
WT+ Palmitate
shSCR+ Palmitate
shFRMD3+ Palmitate
